# A chemoselective and continuous synthesis of *m*-sulfamoylbenzamide analogues

**DOI:** 10.3762/bjoc.13.33

**Published:** 2017-02-16

**Authors:** Arno Verlee, Thomas Heugebaert, Tom van der Meer, Pavel I Kerchev, Frank Van Breusegem, Christian V Stevens

**Affiliations:** 1 Department of Sustainable Organic Chemistry and Technology, Faculty of Bioscience Engineering, Ghent University, Campus Coupure, Coupure Links 653, B-9000 Ghent, Belgium; 2 Department of Plant Systems Biology, VIB, Ghent University, Technologiepark 927, B-9000 Ghent, Belgium; 3 Department of Plant Biotechnology and Bioinformatics, Ghent University, Technologiepark 927, B-9052 Ghent, Belgium

**Keywords:** flow chemistry, medium-throughput synthesis, *m*-sulfamoylbenzamide analogues

## Abstract

For the synthesis of *m-*sulfamoylbenzamide analogues, small molecules which are known for their bioactivity, a chemoselective procedure has been developed starting from *m*-(chlorosulfonyl)benzoyl chloride. Although a chemoselective process in batch was already reported, a continuous-flow process reveals an increased selectivity at higher temperatures and without catalysts. In total, 15 analogues were synthesized, using similar conditions, with yields ranging between 65 and 99%. This is the first automated and chemoselective synthesis of *m*-sulfamoylbenzamide analogues.

## Introduction

**Figure 1:**
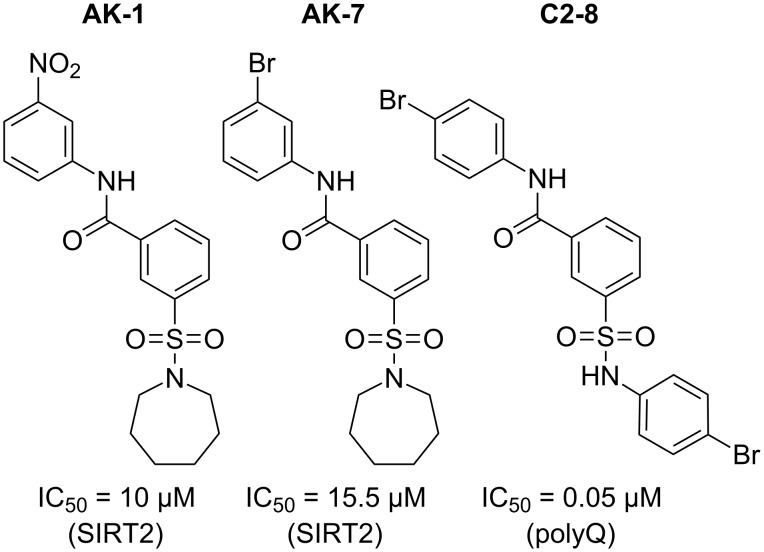
*m*-Sulfamoylbenzamides as Sirtuin 2 inhibitors (SIRT2) or suppressor of polyglutamine aggregation (polyQ).

Small molecules are commonly used for their ability to regulate or assist in different biological processes. Typically, drug development starts with the screening of large libraries of relatively similar compounds, where only milligrams of material are needed for primary testing. Upon identification of a primary hit, the synthetic protocol must then be quickly expanded to tens of grams for early in vivo toxicity studies and hundreds of grams for further toxicology studies and clinical trials [1]. These swiftly changing requirements appear throughout the clinical development of active pharmaceutical ingredients (APIs) and place specific and conflicting burdens on synthetic protocols. An early synthesis must be extremely fast and flexible, as current high-throughput compound screening takes less than one week for a set of 10,000 compounds [2], which is far beyond the current synthetic capabilities. Once a suitable hit is identified on the other hand, the synthetic prerequisites change completely, and a robust and scalable protocol is needed. Over the past few years, flow chemistry has emerged as a potential solution to these conflicting prerequisites [3–11]. Flow processing is suitable for automation, thus allowing the fast synthesis of compound libraries, but as opposed to, e.g., combinatorial chemistry, the developed protocols are directly useful for scale-up. A class of small molecules where these principles can apply for are *m*-sulfamoylbenzamides. These compounds proved to be effective against Huntington’s and Parkinson’s disease [12–14]. They inhibit the Sirtuin 2 (SIRT2) deacetlyse protein (Figure 1, **AK-1**, **AK-7**) resulting in improved motor skills [12–13,15]. Furthermore, *m*-sulfamoylbenzamide analogues (Figure 1, **C2-8**) are able to suppress polyglutamine (polyQ) aggregation [14], which is a major cause of neurodegeneration in Huntington’s disease. Although there are numerous reports available on the study of these analogues, an automated, chemoselective alternative to the synthesis is not yet available.

The most common synthetic approach starts from *m*-(chlorosulfonyl)benzoic acid [15–17]. This synthetic approach is a two-step procedure and therefore needs two subsequent work-up steps, limiting the yield and resulting in a more time-consuming synthetic approach. Yang et al. [18] reported a one-pot synthetic strategy for *m*-sulfamoylbenzamide analogues starting from *m*-(chlorosulfonyl)benzoyl chloride. In this study the difference in reactivity between the sulfonyl and aroyl chloride is exploited resulting in a chemoselective synthesis for these analogues. The yields varied between 46% and quantitative yield, relatively short reaction times were required and dichloromethane was used as solvent.

The coupling of carboxylic acids with amines in flow through a benzotriazole activation [19], or with immobilized reagents as for the synthesis of grossamide [20] is already known. However, we wanted to use *m*-(chlorosulfonyl)benzoyl chloride since this can be synthesized in one single step. Furthermore, acid chlorides show a high reactivity [21] making *m*-(chlorosulfonyl)benzoyl chloride an ideal starting material as was shown by Yang et al. [18]. By transferring this reaction to a multistep flow set-up, we envisioned an improved chemoselectivity. This phenomenon is not unusual for flow chemistry. Typical batch reactions are mixed by stirring; however, perfect homogeneity is not immediately obtained. Ideal mixing conditions can only be achieved with microreactors or micromixers [22]. The small diameters of these microreactors lead to almost ideal mixing conditions [23–26], resulting in an improved chemoselectivity. Furthermore, the use of an automated process leads to the possibility to produce libraries of compounds in a fast manner. In addition, an alternate biocompatible and water miscible solvent would result in a flexible and automated chemoselective synthesis, delivering stock solutions suitable for initial testing at the outlet of the reactor.

## Results and Discussion

### Development of a continuous-flow process

Although a continuous-flow process shows many advantages compared to batch reactions, there are some difficulties which should be overcome or be avoided. A general concern is the clogging of the channels. There are numerous reports about handling solids in flow. For example, the use of ultrasound [27–32] can reduce the particle size of the precipitates, and preventing the clogging of the small channels. A second example is the Coflore agitating cell reactor [32]. This type of reactor uses transverse mixing motions which keeps the solids in suspension, and prevents clogging. The Coflore reactor was successfully used for the synthesis of *N*-iodomorpholinium hydroiodide salt [33]. However, it takes specialized machinery and time to develop a system which can pump slurries. Therefore, a reduction in the formation of solids is preferable. Furthermore, we wanted to avoid the use of dichloromethane as solvent and use a biocompatible and water miscible alternative.

A series of initial batch reactions were performed to evaluate the potential of a chemoselective synthesis as a continuous process. As bench mark, aniline and azepane were used as first and second reagent, respectively. After addition of the first reactant and completion of the reaction (followed by TLC) the second reactant was added. The chemoselectivity was determined by LC–MS analysis.

In the initial screening, tetrahydrofuran (THF) was chosen as solvent (*c*_final_ = 100 mM), however, precipitation of the ammonium salts was unavoidable. The results of this screening did show that the use of catalysts, such as pyridine or dimethylaminopyridine (DMAP), is unnecessary in batch or continuous flow. This is not surprising since a similar result is reported for the reaction of amines with sulfonyl chlorides [34]. Triethylamine was added as base for the capture of hydrogen chloride which is produced during the reaction. Nonetheless, the precipitation of anilinium salts and/or triethylamonium salts could not be avoided in THF, even at lower concentrations (*c*_final_ = 10 mM). Due to the reactivity of the aroyl and sulfonyl chloride, water, DMF or DMSO cannot be used to dissolve the salts. Therefore, acetonitrile (CH_3_CN) was used instead. CH_3_CN is a more polar solvent compared to THF, however, the salts which were formed during the reaction still precipitated (*c*_final_ = 100 mM and 40 mM). At lower concentrations (*c*_final_ = 10 mM), the precipitation of the formed salts was not observed. Furthermore, the chemoselectivity was increased, being 80% for 10 mM and 73% for 100 mM.

### Screening for the optimal chemoselectivity

Since the formation of precipitants can be avoided using CH_3_CN at a final compound concentration of 10 mM, the synthesis can be further optimized in continuous flow. To get the optimal selectivity and reaction conditions, different parameters were screened (residence time/flow rate and reactor temperature). The advantage of the serial use of two microreactors is that two different temperatures can be used. Three solutions were made: F_1_ and F_2_ having a concentration of 40 mM, and F_3_ having a concentration of 20 mM. After addition of the three reaction streams, with the flow rate of F_3_ being twice as high as for F_1_ and F_2_, the final concentration is 10 mM. This corresponds to the end concentration of the selected batch reaction. The results of screening of residence time/flow rate and reactor temperature are presented in Table 1. The optimal conditions and selectivity are obtained for a flow rate of 125 µL/min for starting materials **1** and **2a** and 250 µL/min for reactant **2b**. The temperature for the first microreactor was kept at 20 °C to avoid coupling with the sulfonyl chloride. The second reactor was kept at 40 °C. This increase in temperature enables the coupling with the less reactive sulfonyl chloride, and prevents the use of catalysts.

**Table 1:** Screening results of the different conditions for the best chemoselectivity with aniline and azepane as (F_2_) and (F_3_), respectively.

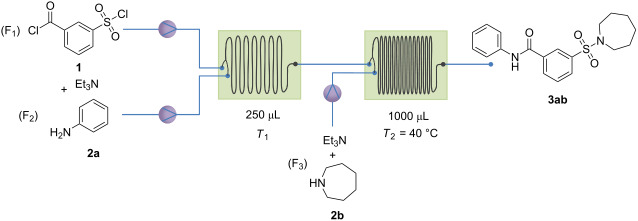				

Run	Flow rate 1 (µL/min)	Flow rate 2 (µL/min)	*T*_1_ (°C)	Chemoselectivity (%)

1	50	100	0	91
2	75	150	0	80

3	25	50	10	89
4	50	100	10	91
5	75	150	10	93
6	100	200	10	92
7	125	250	10	89

8	75	150	20	92
9	100	200	20	93
10	125	250	20	94
batch	–	–	0	80^a^
batch	–	–	20	75^a^

^a^Reaction performed in batch with a final concentration of 10 mM.

With this process, an automated and chemoselective continuous synthesis was obtained for *m*-sulfamoylbenzamide analogues. Furthermore, the chemoselectivity was increased significantly compared to the batch reaction due to the quasi-ideal mixing conditions, and therefore avoiding coupling with the sulfonyl chloride which is less reactive compared to the aroyl chloride [35]. For the reactions in batch, an average chemoselectivity of 80% was obtained while for the synthesis in continuous flow with CH_3_CN the average chemoselectivity is 94%. This indicates that these optimized mixing conditions are crucial for an improved chemoselective synthesis. Interestingly, the temperatures used for the first coupling (*T*_1_ = 20 °C) are substantially higher compared to the batch reactions (0 °C), while the chemoselectivity is still maintained. This effect is also linked to the optimized mixing conditions enabling higher temperatures without losing chemoselectivity, while increasing the reaction rate. This adds also significantly to an increased sustainability of the process since no cooling capacity is required.

This process can be used for a range of *m*-sulfamoylbenzamide analogues (vide infra). However, if the first reagent (F_2_) is a secondary amine, the chemoselectivity decreases substantially when the current process is used. Secondary amines are more nucleophilic as compared to primary amines, resulting in a higher percentage of sulfonylation. To improve the chemoselectivity when using secondary amines, an additional screening was performed with azepane and aniline as first and second reagent, respectively. Initially, we tried to increase the selectivity by decreasing the temperature. Unfortunately, the reaction mixtures obtained showed the presence of several side products, and the decrease in temperature did not appear to result in a substantial increase in chemoselectivity. Therefore, it was decided to first optimize the chemoselectivity for compound **4** (Table 2). This simplified the reaction mixture substantially. The temperature was kept at −15 °C and the final concentration of compound **4** was varied between 20 mM and 5 mM. By decreasing the concentration, the chemoselectivity increased substantially from 45% for 40 mM to 89% for 5 mM.

**Table 2:** Screening results of the different conditions for the best chemoselectivity with azepane as (F_2_).

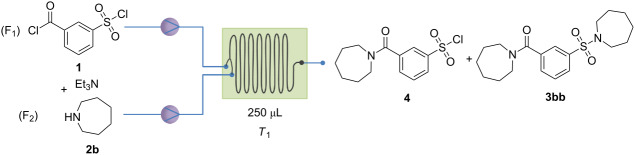					

Run	Flow rate (µL/min)	Concentration F_1_ and F_2_ (mM)	Final concentration (mM)	**4** (%)	**3bb** (%)

1	50	40	20	29	36
2	100	40	20	29	36
3	200	40	20	28	36

4	50	20	10	37	32
5	100	20	10	60	20
6	150	20	10	47	27

7	100	10	5	63	19
8	150	10	5	71	15
9	200	10	5	64	18

10	200	5	2.5	67	17
11	400	5	2.5	74	13
12	600	5	2.5	82	9

Using the lower substrate concentration, an optimization of the second reaction step was performed, using azepane as the first and aniline as the second reactant. The concentrations used were 5 mM for F_1_ and F_2_ and 2.5 mM for F_3_. This leads to a final concentration of 1.25 mM. However, due to the increased flow rate, the second coupling step with aniline could not reach full conversion. Even by increasing the temperature for this step up to 75 °C, full conversion was not obtained. Therefore, DMAP was used as a base instead of triethylamine in F_3_. DMAP serves both as a base and catalyst for the reaction with the sulfonyl chloride group. The temperature in the second reation chip was kept at 75 °C and by using DMAP as a base, full conversion was obtained. The effect of the temperature and the flow rate were evaluated and the results are shown in Table 3. The highest chemoselectivity (82%) was obtained for a flow rate of 500 µL/min for F_1_ and F_2_ and 1000 µL/min for F_3_ at a temperature of 0 °C and 75 °C in chip 1 and chip 2, respectively. It should be noted that not the reaction temperature, but rather the substrate concentration is the main variable determining chemoselectivity (compare Table 3, entries 2 and 5). The chemoselectivity in flow was again higher compared to the batch conditions due to quasi-ideal mixing conditions.

**Table 3:** Screening results of the different conditions for the best chemoselectivity with azepane and aniline as (F_2_) and (F_3_), respectively.

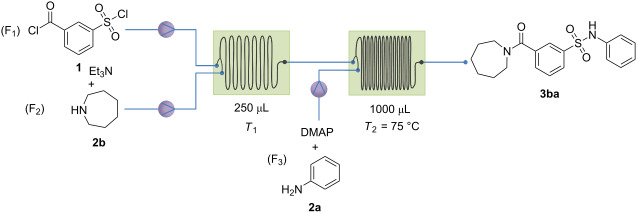				

Run	Flow rate 1 (µL/min)	Flow rate 2 (µL/min)	*T*_1_ (°C)	Chemoselectivity (%)

1	300	600	−15	39
2	400	800	−15	79
3	500	1000	−15	57

4	300	600	0	53
5	400	800	0	80
6	500	1000	0	82
7	600	1200	0	52

8	200	400	10	41
9	300	600	10	50
10	400	800	10	40
batch	–	–	0	59^a^

^a^Reaction performed in batch with a final concentration of 1.25 M.

### Medium-throughput synthesis

To evaluate the flexibility of both processes, a range of molecules were synthesized on small scale. In total, 49 molecules could be readily used for a medium-throughput screening for pharmaceutical applications. The chemoselectivity was measured by LC–MS and is presented in Table 4. The chemoselectivity varied between 50 and 99%. Apart from the reactions involving 3-fluoroaniline, the chemoselectivity was above 70% for primary amines and above 60% for secondary amines. The side products which are being formed are the double substituted analogues **3aa**, **3bb**, **3cc**, **3dd**, **3ee, 3ff** or **3ee** depending on the amines which were used. As such, we synthesized these compounds (chemoselectivity >99%) so that they can function as a negative control in the direct screening, to exclude false positives. Synergistic effects were not taken into account but, the screening of these analogues should already give a good indication which compounds are of interest.

#### Medium throughput synthesis if F_2_ are primary amines

Between each sample a washing step with CH_3_CN was included to eliminate any side reaction of undesired amines in the system. For the washing step, a flow rate of 1000 µL/min was applied for a duration of 4 minutes. This implements a total washing volume of 12 mL, which is 8 times the total volume of the flow system. The equilibration time was 11.5 minutes and the collecting time 1.5 minutes resulting in a reaction time of 13 minutes. The volume collected for each sample was 750 µL, and a total reaction time, including the washing step, of 17 minutes is required. On a 24 h basis, a total of 84 compounds can be synthesized in continuous flow, and used for a medium-throughput screening with primary amines as first reactant. The final concentration of each sample was 10 mM and can be diluted with a factor 100 resulting in a concentration of 100 µM. In each sample, only 1% (v/v) of CH_3_CN would be present.

#### Medium throughput synthesis if F_2_ are secondary amines

If the first reagent is a secondary amine, the washing step remains the same and the volume collected was 1000 µL. The equilibration time was 5 minutes and the collecting time together with the equilibration time was 5 minutes and 20 seconds. The total reaction time, including the washing step, was approximately 10 minutes. This leads to 144 compounds on a daily basis. The final concentration of each sample was 1.25 mM and can be diluted with a factor 12.5 resulting in a concentration of 100 µM. In each sample 8% (v/v) of CH_3_CN would be present. The next step is to produce these compounds on a larger scale. From Table 4, 15 analogues were chosen and produced on a larger scale (vide infra).

**Table 4:** Chemoselectivity (%) of the medium-throughput synthesis in continuous flow.

		(F_3_)	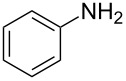 **2a**	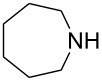 **2b**	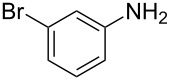 **2c**	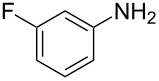 **2d**	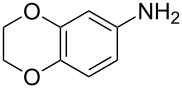 **2e**	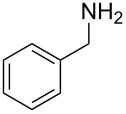 **2f**	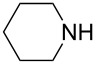 **2g**
(F_2_)									

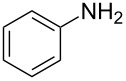 **2a**			99 **3aa**	94 **3ab**	95 **3ac**	64 **3ad**	95 **3ae**	83 **3af**	94 **3ag**
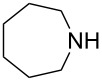 **2b**			82 **3ba**	99 **3bb**	83 **3bc**	77 **3bd**	76 **3be**	70 **3bf**	74 **3bg**
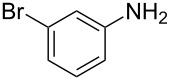 **2c**			83 **3ca**	94 **3cb**	99 **3cc**	64 **3cd**	94 **3ce**	84 **3cf**	97 **3cg**
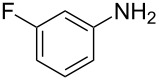 **2d**			64 **3da**	59 **3db**	53 **3dc**	99 **3dd**	58 **3de**	68 **3df**	85 **3dg**
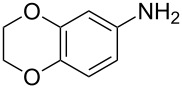 **2e**			93 **3ea**	94 **3eb**	94 **3ec**	63 **3ed**	99 **3ee**	86 **3ef**	94 **3eg**
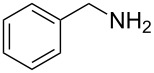 **2f**			77 **3fa**	73 **3fb**	71 **3fc**	50 **3df**	77 **3fe**	99 **3ff**	72 **3fg**
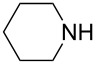 **2g**			68 **3ga**	69 **3gb**	83 **3gc**	63 **3gd**	60 **3ge**	61 **3gf**	99 **3gg**

#### Synthesis of a small library in continuous flow

The use of flow chemistry facilitated greatly the synthesis of an extended library of compounds. Different *m*-sulfamoylbenzamide analogues were synthesized in continuous flow. From Table 4, 15 analogues were produced on a larger scale to exemplify the direct scalability of the developed protocol. For these reactions the required amount of product was aimed at 100–200 mg which took about 3 hours of production. Compound **3cb**, which corresponds with **AK-7**, was also produced on gram scale which took approximately 24 hours. Table 5 shows 15 analogues synthesized in continuous flow. The chemoselectivity varied between 83 and 97%, the remaining 17–3% were symmetrical *m*-sulfamoylbenzamides. After work-up and purification the yield was between 70 and 99%. These results indicate that both processes are applicable to a large variety of *m-*sulfamoylbenzamides.

**Table 5:** Library of 15 *m-*sulfamoylbenzamide analogues synthesized in continuous flow.

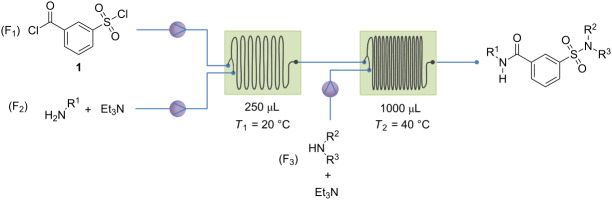					

Compound	(F_2_)	(F_3_)	Chemoselectivity (%)	Yield (%)	Quantity (mg)

**3aa**	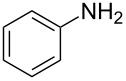	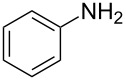	–	95	140
**3ab**		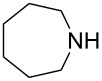	94	75	197
**3ac**		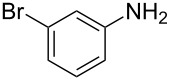	95	78	94
**3ae**		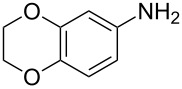	95	76	92
**3ag**		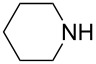	94	74	88

**3ca**	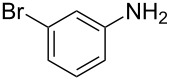	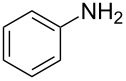	83	70	83
**3cb**		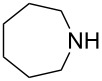	94 87^a^	78 80	93 2447
**3cc**		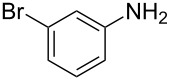	–	99	97
**3ce**		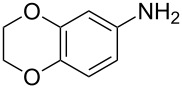	94	81	77
**3cg**		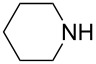	97	77	96

**3ea**	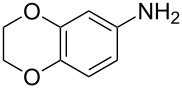	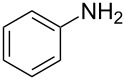	93	80	96
**3eb**		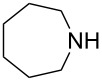	94	78	94
**3ec**		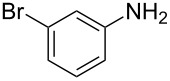	94	72	87
**3ee**		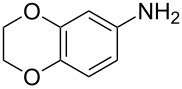	–	98	117
**3eg**		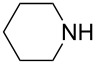	94	77	93

^a^Reduced chemoselectivity due to leakage during the reaction.

## Conclusion

A chemoselective, automated process is developed for the synthesis of *m-*sulfamoylbenzamide analogues. The used solvent is acetonitrile and the reactions in continuous flow showed an increased chemoselectivity compared to the batch reactions due to the ideal mixing conditions. Using secondary amines, a decrease in substrate concentration was essential to selectively obtain amides over sulfonamides. It was shown that the procedure can easily be used for the synthesis of a compound library suitable for initial screening; and that the optimized synthetic conditions are directly transferrable should the resulting hits be needed in gram-scale for further evaluation.

## Experimental

### General

**Figure 2:**
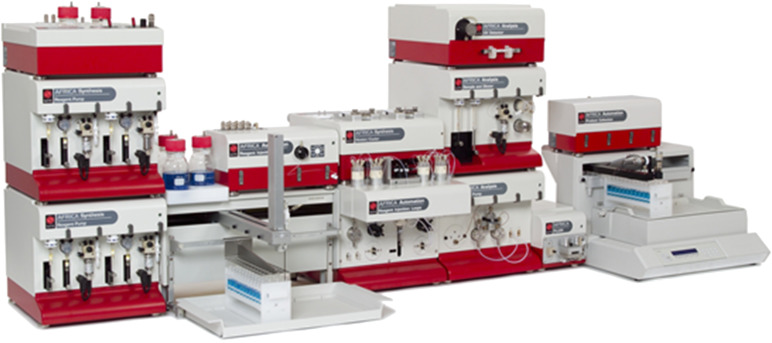
Syrris AFRICA system.

All chemicals were purchased by either Sigma-Aldrich or TCI chemicals. Commercially available products were used without additional purification. NMR spectra were recorded at 400 MHz (^1^H) and 100 MHz (^13^C) in CDCl_3_ with tetramethylsilane as internal standard or DMSO-*d*_6_ on a Bruker Avance III Nanobay 400 MHz spectrometer at room temperature. The automated continuous synthesis was conducted using a commercially available continuous-flow system (syrris AFRICA, Figure 2).

### Representative procedure for *m*-sulfamoylbenzamide analogues

a) Continuous process with primary amines as F_1_: Triethylamine and a primary amine (F_1_) were dissolved in acetonitrile (*c* = 40 mM), *m*-chlorosulfonylbenzoyl chloride (F_2_) was dissolved in the same solvent in a separate volumetric flask (*c* = 40 mM). A third solution was prepared with triethylamine and the second reactant (F_3_) (*c* = 20 mM). The flow process is presented in Table 1; reactants **1** and **2x** were mixed together in reactor 1 at 20 °C at a flow rate of 125 µL/min. The reaction mixture was then pumped to reactor 2, which was kept at 40 °C. The third reactant (**2y**) was then added at a flow rate of 250 µL/min. The residence times were 1 min and 2 min, respectively. Once the mixture passed both reactors, the final compound concentration was 10 mM and could be used as a stock solution for initial screening. For the larger scale experiments, the work-up procedure was similar to a batch reaction. The solvent was removed in vacuo and the remaining oil was dissolved in diethyl ether. It was subsequently washed with a hydrogen chloride solution of 1 M and with a saturated sodium bicarbonate solution. The organic phase was dried with MgSO_4_, the solvent was evaporated in vacuo*,* and the residue was purified by either preparative thin-layer chromatography or by recrystallization.

b) Continuous process with secondary amines as F_1_: Triethylamine and a secundary amine (F_1_) were dissolved in acetonitrile (*c* = 5 mM), *m*-chlorosulfonylbenzoyl chloride (F_2_) was dissolved in acetonitrile in a second volumetric flask (*c* = 5 mM). A third solution was prepared with dimethylaminopyridine (DMAP) and the second reactant (F_3_) (*c* = 2.5 mM). The flow process is presented in Table 3; reactants **1** and **2x** were mixed together in reactor 1 at 0 °C at a flow rate of 500 µL/min. The reaction mixture was then pumped to reactor 2, which was kept at 75 °C. The third reactant (**2y**) was then added at a flow rate of 1000 µL/min. The residence times were 0.25 min and 0.5 min, respectively. Once the mixture passed both reactors, the final compound concentration was 1.25 mM and could be used as a stock solution for screening.

## Supporting Information

File 1 Experimental part.
