# Supplementary material for: A chemoselective and continuous synthesis of m-sulfamoylbenzamide analogues
Source: Beilstein J Org Chem. 2017 Feb 16;13:303–12. doi: 10.3762/bjoc.13.33 (PMC5331323; doi:10.3762/bjoc.13.33)
Supplement: File 1 — Experimental part. [file Beilstein_J_Org_Chem-13-303-s001.pdf]

Supporting Information

for

A chemoselective and continuous synthesis of *m*-sulfamoylbenzamide analogues

Arno Verlee[1], Thomas Heugebaert[1], Tom van der Meer[2,3], Pavel I. Kerchev[2,3], Frank Van Breusegem[2,3] and Christian V. Stevens[1,*]

Address: [1]Department of Sustainable Organic Chemistry and Technology, Faculty of Bioscience Engineering, Ghent University, Campus Coupure, Coupure Links 653, B-9000 Ghent, Belgium, [2]Department of Plant Systems Biology, VIB, Ghent University, Technologiepark 927, B-9000 Ghent, Belgium and Department of Plant Biotechnology and Bioinformatics, Ghent University, Technologiepark 927, B-9052 Ghent, Belgium

Email: Christian V. Stevens - Chris.Stevens@UGent.be

*Corresponding author

Experimental part

Synthesis of compound ***1***

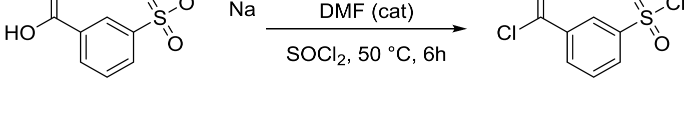

To a suspension of *m*-sulfobenzoic acid monosodium salt (10 g, 44.6 mmol) in thionyl chloride (200 mL), a catalytic amount of dimethylformamide (1 mL, 13.4 mmol) was added. Subsequently, the reaction was heated to 50 °C. After 6 hours, the reaction

mixture was filtered and the filtrate was concentrated in vacuo resulting in the desired final product. The compound was used without further purification.

Yellow oil (95%); ^{1}H-NMR (400 MHz, $CDCl_3$): δ 8.75 (m, 1H), 8.50 (ddd, 1H, *J*= 7.9, 1.6, 1.1 Hz), 8.31-8.28 (ddd, 1H, *J*= 8.0, 1.6, 1.1 Hz), 7.80 (dd, 1H *J*= 8.0 Hz); ^{13}C-NMR (100 MHz, $CDCl_3$): δ 166.5 (C), 145.3 (C), 137.0 (CH), 135.0 (CH), 132.8 (C), 130.9 (CH), 129.4 (CH); IR (neat): *v* = 3076 (w), 1753 (s), 1593 (m), 1377 (s), 1159 (s), 1082 (m), 997 (m), 930 (s), 806 (m), 689 (s), 660 (s), 571 (s) cm^{-1}.

Compound **3aa**

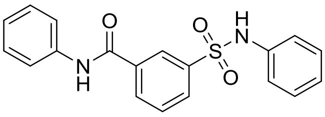

White solid (no further purification; 140 mg 95%); m.p. = 155 - 156 °C; ^{1}H-NMR (400 MHz, DMSO-d_6): δ 10.48 (s, 1H), 10.41 (s, 1H), 8.34 (dd, 1H, *J*= 1.7 Hz), 8.18 (ddd, 1H, *J*= 7.9, 1.7 Hz), 7.92 (ddd, 1H, *J*= 7.8, 1.6 Hz), 7.75 (d, 2H, *J*= 7.8 Hz), 7.71 (dd, 1H, *J*= 7.9 Hz), 7.37 (dd, 2H, *J*= 7.8 Hz), 7.24 (dd, 2H, *J*= 7.8 Hz), 7.16 – 7.09 (m, 3H), 7.03 (dd, 1H, *J*= 7.3 Hz); ^{13}C-NMR (100 MHz, DMSO-d_6): δ 164.4 (C), 140.3 (C), 139.1 (C), 137.8 (C), 136.2 (C), 132.1 (CH), 129.8 (CH), 129.7 (CH), 129.6 (CH), 129.0 (CH), 126.5 (CH), 124.6 (CH), 124.4 (CH), 120.8 (CH), 120.6 (CH); IR (neat): *v*= 3379 (w), 3136 (w), 3074 (w), 1655 (s), 1599 (m), 1533 (s), 1493 (m), 1445 (m), 1327 (m), 1159 (s), 1080 (m), 922 (m), 746 (s), 691 (s), 556 (s), 513 (m) cm^{-1}; MS: m/z= 353.0 $[M+H]^+$.

Compound **3ab**

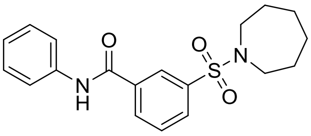

White crystals (recrystallization in methanol; 197 mg, 75%); m.p. = 160 – 161 °C; ^{1}H-NMR (400 MHz, $CDCl_3$): δ 8.26 (dd, 1H, *J*= 1.4 Hz), 8.13 (ddd, 1H, *J*= 1.3, 7.8 Hz),

7.99 (s, br, 1H) 7.98 (ddd, 1H, *J*= 1.3, 7.7 Hz), 7.70 – 7.65 (m, 3H), 7.45 – 7.39 (m, 2H), 7.24 – 7.19 (m, 1H), 3.33 (t, 4H, *J*= 5.9 Hz), 1.75 (m, 4H), 1.66 – 1.60 (m, 4H); ^{13}C-NMR (100 MHz, $CDCl_3$): δ 164.9 (C), 140.4 (C), 138.0 (C), 136.6 (C), 131.9 (CH), 130.1 (CH), 129.5 (CH), 129.4 (CH), 125.5 (CH), 125.3 (CH), 121.0 (CH), 48.7 (CH_2), 29.5 (CH_2), 27.2 (CH_2); IR (neat): *v*= 3311 (w), 2918 (w), 2848 (w), 1651 (m), 1597 (m), 1530 (s), 1441 (m), 1329 (s), 1153 (s), 1038 (m), 743 (s), 687 (s), 565 (s), 513 (s) cm^{-1}; MS: *m/z*= 359.1 $[M+H]^+$.

Compound **3ac**

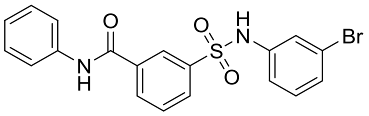

White-yellow solid (recrystallization in methanol; 94 mg, 78%); m.p. = 203 – 204 °C; ^{1}H-NMR (400 MHz, DMSO-d_6): δ 10.53 (s, 1H), 10.45 (s, 1H), 8.39 (dd, 1H, *J*= 1.6 Hz), 8.24-8.22 (m, 1H), 7.99-7.96 (m, 1H), 7.81-7.79 (m, 2H), 7.76 (dd, 1H, *J*= 7.9 Hz), 7.44-7.40 (m, 2H), 7.29-7.27 (m, 2H), 7.17-7.15 (m, 2H), 7.11-7.06 (m, 1H); ^{13}C-NMR (100 MHz, $CDCl_3$): δ 164.5 (C), 140.4 (C), 139.2 (C), 136.3 (C), 132.2 (CH), 129.9 (CH), 129.8 (CH), 129.7 (CH), 129.1 (CH), 126.6 (C), 124.8 (CH), 124.5 (CH), 120.96 (CH), 120.7 (CH); IR (neat): *v* = 3345 (m), 3127 (w), 1645 (s), 1597 (m), 1524 (s), 1447 (s), 1393 (m), 1339 (m), 1263 (m), 1161 (s), 1124 (m), 951 (s), 878 (m), 762 (m), 677 (s), 588 (s), 507 (m) cm^{-1}; MS: *m/z* = 430.9 $[M+H]^+$; 433.0 $[M+H]^+$.

Compound **3ae**

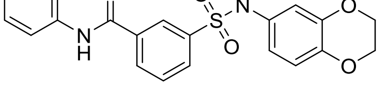

White solid (column chromatography: 55% Ethyl acetate:hexane; 92 mg, 76%); m.p. = 165 – 166 °C; ^{1}H-NMR (400 MHz, $CDCl_3$): δ 8.30 (dd, 1H, *J*= 1.6 Hz), 8.26 (s, br, 1H), 8.08-8.05 (m, 1H), 7.78-7.76 (m, 1H), 7.62-7.59 (m, 1H), 7.48 (dd, 1H, *J*= 7.9

Hz), 7.28-7.23 (m, 2H), 7.26-7.25 (m, 1H), 7.16-7.11 (m, 1H), 6.69 (d, 1H, *J*= 8.7 Hz), 6.67 (d, 1H, *J*= 2.5 Hz), 6.53 (dd, 1H, *J*= 8.5, 2.5 Hz), 4.17-4.16 (m, 4H); ^{13}C-NMR (100 MHz, $CDCl_3$): δ 164.2 (C), 143.7 (C), 142.2 (C), 139.5 (C), 137.5 (C), 135.9 (C), 134.2 (CH), 132.2 (CH), 130.3 (CH), 129.6 (CH), 129.1 (C), 129.1 (CH), 125.4 (CH), 125.0 (CH), 120.7 (CH), 117.6 (CH), 117.0 (CH), 113.1 (CH), 64.2 (CH_2), 64.2 (CH_2); IR (neat): *v*= 3368 (w), 3138 (w), 1649 (m), 1599 (m), 1533 (m), 1503 (m), 1445 (m), 1339 (m), 1300 (m), 1258 (m), 1157 (s), 1065 (m), 883.4 (m), 758 (m), 681 (s), 590 (s), 554 (s), 503 (m) cm^{-1}; MS: *m/z*= 411.0 $[M+H]^+$.

Compound **3ag**

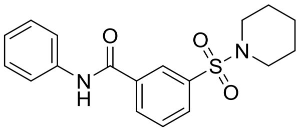

White-yellow solid (recrystallization in methanol; 88 mg, 74%); m.p. = 158 – 159 °C; ^{1}H-NMR (400 MHz, $CDCl_3$): δ 8.20 (dd, 1H, *J*= 1.6 Hz), 8.16-8.13 (m, 1H), 8.01 (s, br, 1H), 7.93-7.90 (m, 1H), 7.70-7.65 (m, 3H), 7.42-7.37 (m, 2H), 7.21-7.17 (m, 1H), 3.02 (t, 4H, *J*= 5.4 Hz), 1.64 (tt, 4H, *J*= 5.6 Hz), 1.45-1.40 (m, 2H); ^{13}C-NMR (100 MHz, $CDCl_3$): δ 164.2 (C), 137.5 (C), 137.2 (C), 136.2 (C), 131.7 (CH), 130.5 (CH), 129.8 (CH), 129.2 (CH), 125.6 (CH), 125.1 (CH), 120.1 (CH), 47.0 (CH_2), 25.1 (CH_2), 23.4 (CH_2); IR (neat): *v*= 3321 (w), 2922 (w), 2851 (w), 1653 (s), 1597 (m), 1521 (s), 1323 (s), 1260 (m), 1161 (s), 1092 (m), 1053 (m), 932 (s), 812 (m), 773 (m), 745 (s), 681 (s), 608 (s), 579 (s) cm^{-1}; MS: *m/z*= 345.0 $[M+H]^+$.

Compound **3ca**

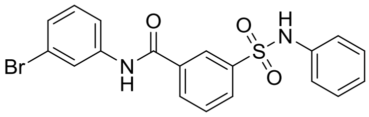

White solid (Column chromatography: 55% ethyl acetate:hexane; 83 mg, 70%); m.p. = 139 – 140 °C; ^{1}H-NMR (400 MHz DMSO-*d*$_6$): δ 10.62 (s, 1H), 10.42 (s, 1H), 8.34

(dd, 1H, *J*=1.6 Hz), 8.19-8.17 (m, 1H), 8.08-8.07 (m, 1H), 7.96-7.93 (m, 1H), 7.75-7.70 (m, 2H), 7.35-7.33 (m, 2H), 7.27-7.22 (m, 2H), 7.12-7.10 (m, 2H), 7.06-7.02 (m, 1H); ^{13}C-NMR (100 MHz, DMSO-d$_6$): δ 164.7 (C), 140.8 (C), 140.5 (C), 137.8 (C), 135.9 (C), 132.3 (CH), 131.2 (CH), 130.1 (CH), 130.1 (CH), 129.7 (CH), 127.1 (CH), 126.6 (CH), 124.8 (CH), 123.2 (CH), 121.9 (C), 120.7 (CH), 119.7 (CH); IR (neat): *v* = 3377 (w), 3167 (w), 1659 (s), 1593 (m), 1528 (m), 1412 (m), 1346 (m), 1161 (s), 1076 (m), 918 (m), 779 (s), 748 (s), 696 (s), 588 (s), 544 (s), 466 (m) cm^{-1}; MS: *m/z* = 431.0 [M+H]$^+$, 433.0 [M+H]$^+$

Compound **3cb**

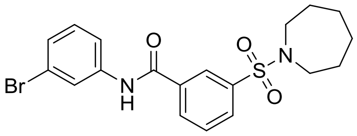

White crystals (recrystallization in methanol; 93 mg, 78%); m.p. = 152 – 153 °C ^{1}H-NMR (400 MHz, DMSO-d_6): δ 8.25 (dd, 1H, *J*= 1.6 Hz), 8.14-8.10 (m, 2H), 8.00-7.96 (m, 2H), 7.68 (dd, 1H, *J*= 7.8 Hz), 7.61-7.59 (m, 1H), 7.35-7.33 (m, 1H), 7.29 (d, 1H, *J*= 7.8 Hz), 3.32 (t, 4H, *J*= 5.9 Hz), 1.78-1.71 (m, 4H), 1.64-1.60 (m, 4H); ^{13}C-NMR (100 MHz, DMSO-d$_6$): δ 164.3 (C), 140.4 (C), 138.8 (C), 135.7 (C), 131.4 (CH), 130.4 (CH), 130.0 (CH), 129.9 (CH), 128.0 (CH), 125.0(CH), 123.4 (CH), 122.8 (C), 118.8 (CH), 48,4 (CH_2), 29.1 (CH_2), 26.9 (CH_2); IR (neat): *v*= 3292 (w), 3075 (w), 2934 (w), 2853 (w) 1651 (s), 1587 (m), 1414 (m), 1332 (s),1159 (s), 876 (m), 773 (s), 687 (s), 582 (s), 567 (s) cm^{-1}; MS: *m/z*= 437.0 [M+H]$^+$, 439.0 [M+H]$^+$.

Compound **3cc**

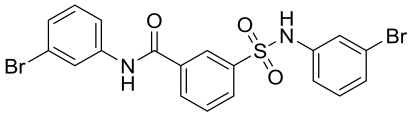

Yellow solid (no further purification; 118 mg, 99%); m.p. = 188 – 189 °C; ^{1}H-NMR (400 MHz, DMSO-d$_6$): δ 10.71 (s, 1H), 10.63 (s, 1H), 8.36 (dd, 1H, *J*= 1.7 Hz), 8.22-

8.20 (m, 1H), 8.08-8.06 (m, 1H), 7.97 (ddd, 1H, *J*= 7.9, 1.7, 1.0 Hz), 7.75 (dd, 1H, *J*= 7.7 Hz), 7.75-7.72 (m, 1H), 7.37-7.33 (m, 2H), 7.28-7.27 (m, 1H), 7.23-7.19 (m, 2H), 7.14-7.12 (m, 1H); 13C-NMR (100 MHz, DMSO-d_6): 164.6 (C), 140.8 (C), 140.1 (C), 139.6 (C), 136.0 (C), 132.6 (CH), 131.8 (CH), 131.2 (CH), 130.3 (CH), 130.0 (CH), 127.4 (CH), 127.1 (CH), 126.6 (CH), 123.2 (CH), 122.6 (CH), 122.3 (C), 121.9 (C), 119.7 (CH), 119.1 (CH); IR (neat): *v*= 3375 (w), 3146 (w), 1657 (m), 1589 (m), 1526 (m), 1477 (m), 1335 (m), 1159 (s), 1072 (m), 883 (m), 777 (s), 707 (m), 590 (s), 436 (m) cm^{-1}; MS: *m/z*= 508.8 $[M+H]^+$, 510.9 $[M+H]^+$.

Compound **3ce**

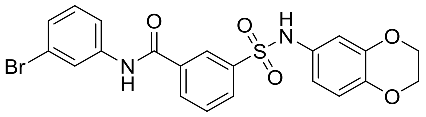

White-brown solid (column chromatography: 55% ethyl acetate:hexane; 97 mg, 81%); m.p. = 145 – 146 °C; ^{1}H-NMR (400 MHz, DMSO-d_6): δ 10.62 (s, 1H), 10.10 (s, 1H), 8.29 (dd, 1H, *J*= 1.6 Hz), 8.20-8.17 (m, 1H), 8.08-8.07 (m, 1H), 7.90 (ddd, 1H, *J*= 7.9, 1.6, 1.0 Hz), 7.76-7.71 (m, 2H), 7.37-7.31 (m, 2H), 6.71 (d, 1H, *J*= 8.6 Hz), 6.58 (d, 1H, *J*= 2.4 Hz), 6.54 (dd, 1H, *J*= 8.6, 2.5 Hz), 4.17-4.13 (m, 4H); ^{13}C-NMR (100 MHz, DMSO-d_6): δ 164.8 (C), 143.7 (C), 141.2 (C), 140.9 (C), 140.4 (C), 135.8 (C), 132.2 (CH), 131.2 (CH), 130.9 (C), 130.1 (CH), 130.0 (CH), 127.1 (CH), 126.6 (CH), 123.2 (CH), 121.9 (C), 119.7 (C), 117.8 (CH), 115.1 (CH), 111.0 (CH), 64.5 (CH_2), 64.3 (CH_2); IR (neat): *v* = 3333 (w), 3118 (w), 2965 (w), 2878 (w), 1649 (m), 1593 (m), 1510 (s), 1333 (m), 1254 (m), 1157 (s), 1070 (m), 980 (m), 856 (m), 775 (s), 675 (s), 583 (s), 490 (m) cm^{-1}; MS: *m/z* = 488.9 $[M+H]^+$, 490.9 $[M+H]^+$.

Compound **3cg**

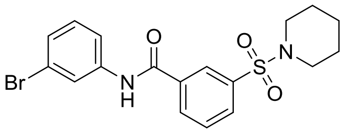

White crystals (recrystallization in methanol; 92 mg, 77%); m.p. = 167 – 168 °C; ^{1}H-NMR (400 MHz, $CDCl_3$): δ 8.41 (s, br, 1H), 8.20 (dd, 1H, *J*= 1.6 Hz), 8.16-8.13 (m, 1H), 7.97 (dd, 1H, *J*= 1.7 Hz), 7.90-7.87 (m, 1H), 7.67 (dd, 1H, *J*= 7.8 Hz), 7.61 (d, 1H, *J*= 8.0 Hz), 7.32-7.29 (m, 1H), 7.24 (dd, 1H, *J*= 8.0 Hz), 2.99 (t, 4H, *J*= 5.4 Hz), 1.61 (tt, 4H, *J*= 5.6 Hz), 1.41 (tt, 2H, *J*= 5.7 Hz); ^{13}C-NMR (100 MHz, $CDCl_3$): δ 164.4 (C), 138.9 (C), 137.0 (C), 135.8 (C), 132.0 (CH), 130.6 (CH), 130.4 (CH), 129.8 (CH), 127.9 (CH), 125.7 (CH), 123.5 (CH), 122.7 (C), 119.1 (CH), 47.0 (CH_2), 25.1 (CH_2), 23.4 (CH_2); IR (neat): *v*= 3296 (w), 2947 (w), 2845 (w), 1653 (m), 1589 (m), 1520 (m), 1476 (m), 1414 (m), 1337 (s), 1294 (m), 1161 (s), 1051 (m), 935 (s), 775 (s), 685 (s), 571 (s), 486 (m) cm^{-1}; MS: *m/z*= 423.0 $[M+H]^+$; 425.0 $[M+H]^+$.

Compound **3ea**

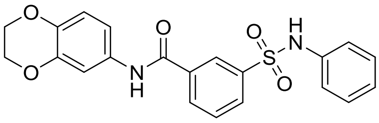

White-brown solid (column chromatography: 65% ethyl acetate: hexane; 96 mg, 80%); m.p. = 192 – 193 °C; ^{1}H-NMR (400 MHz, $CDCl_3$): δ 8.20 (dd, 1H, *J*= 1.5 Hz), 8.08-8.05 (m, 1H), 7.87-7.84 (m, 1H), 7.68 (s, br, 1H), 7.54 (dd, 1H, *J*= 7.9 Hz), 7.29-7.25 (m, 2H), 7.23 (d, 1H, *J*= 2.0 Hz), 7.19-7.15 (m, 1H), 7.11-7.08 (m, 1H), 6.98 (dd, 1H, *J*= 8.8, 2.2 Hz), 6.84 (d, 1H, *J*= 8.7 Hz), 4.28-4.24 (m, 4H); ^{13}C-NMR (100 MHz, $CDCl_3$): δ 164.0 (C), 143.5 (C), 141.0 (C), 139.5 (C), 136.1 (C), 136.0 (C), 132.1 (CH), 131.0 (C), 130.1 (CH), 129.5 (CH), 129.4 (CH), 125.9 (CH), 125.4 (CH), 122.4 (CH), 117.3 (CH), 114.3 (CH), 110.5 (CH), 64.4 (CH_2), 64.3 (CH_2); IR (neat): *v* = 3389 (w), 3076 (w), 2889 (w), 1639 (s), 1597 (m), 1506 (s), 1429 (m), 1342 (m), 1157

(s), 1065 (s), 920 (s), 889 (s), 767 (m), 729 (m), 679 (s), 543 (m) cm^{-1}; MS: *m/z* = 411.0 $[M+H]^+$.

Compound **3eb**

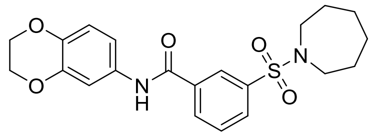

White crystals (recrystallization in methanol; 94 mg, 78%); m.p. = 115 – 116 °C; ^{1}H-NMR (400 MHz, $CDCl_3$): δ 8.2 (dd, 1H, *J*= 1.6 Hz), 8.09-8.07 (m, 1H), 7.95-7.92 (m, 1H), 7.83 (s, br, 1H), 7.63 (dd, 1H, *J*= 7.8 Hz), 7.29 (d, 1H, *J*= 1.9 Hz), 7.03 (dd, 1H, *J*= 8.7, 2.1 Hz), 6.86 (d, 1H, *J*= 8.7 Hz), 4.28-4.25 (m, 4H), 3.29 (t, 4H, *J*= 5.9 Hz), 1.75-1.69 (m, 4H), 1.61-1.57 (m, 4H) ^{13}C-NMR (100 MHz, $CDCl_3$): δ 164.0 (C), 143.6 (C), 141.0 (C), 140.3 (C), 136.2 (C), 131.3 (C), 131.1 (CH), 129.8 (CH), 129.7 (CH), 124.9 (CH), 117.4 (CH), 114.1 (CH), 110.3 (CH), 64.4 (CH_2), 64.3 (CH_2), 48.4 (CH_2), 29.1 (CH_2), 26.9 (CH_2); IR (neat): *v* = 3302 (w), 2920 (w), 2853 (w), 1641 (m), 1607 (s), 1506 (s), 1412 (s), 1328 (s), 1279 (s), 1152 (s), 1069 (s), 920 (m), 862 (m), 733 (m), 577 (s) cm^{-1}; MS: *m/z* = 417.0 $[M+H]^+$.

Compound **3ec**

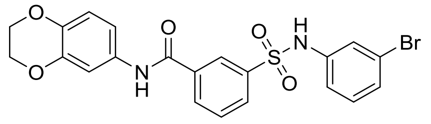

White-yellow solid (column chromatography: 60% ethyl acetate: hexane; 87 mg, 72%); m.p. = /; ^{1}H-NMR (400 MHz, $CDCl_3$): δ 8.52 (s, br, 1H), 8.47 (m, 1H), 8.04-8.01 (m, 1H), 7.77-7.74 (m, 1H), 7.41 (dd, 1H, *J*= 7.8 Hz), 7.32-7.31 (m, 1H), 7.19-7.16 (m, 2H), 7.05-7.02 (m, 2H), 7.00-6.97 (m, 1H), 6.74 (d, 1H, *J*= 8.7 Hz), 4.18-4.17 (m, 4H); ^{13}C-NMR (100 MHz, $CDCl_3$): δ 164.3 (C), 143.4 (C), 141.0 (C), 139.6 (C), 138.4 (C), 136.0 (C), 132.1 (CH), 131.0 (C), 130.6 (CH), 130.1 (CH), 129.4 (CH), 128.2 (CH), 125.6 (CH), 124.6 (CH), 122.7 (C), 120.3 (CH), 117.2 (CH), 114.6 (CH), 110.8

(CH), 64.4 (CH_2), 64.3 (CH_2); IR (neat): *v* = 3271 (w), 2930 (w), 1649 (m), 1593 (m), 1506 (s), 1477 (m), 1333 (m), 1283 (m), 1159 (s), 1066 (m), 921 (m), 887 (m), 736 (m), 682 (m), 586 (m), cm^{-1}; MS: *m/z* = 488.9 $[M+H]^+$; 490.9 $[M+H]^+$

Compound **3ee**

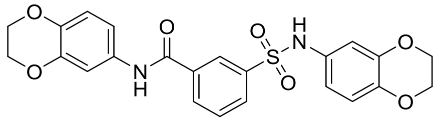

Brown solid (no further purification; 117 mg, 98%); m.p. = /; ^{1}H-NMR (400 MHz, $CDCl_3$): δ 8.25 (dd, 1H, *J*= 1.5 Hz), 8.09-8.05 (m, 1H), 8.01 (s, br, 1H) 7.80-7.78 (m, 1H), 7.76 (s, br, 1H), 7.50 (dd, 1H, *J*= 7.8 Hz), 7.22 (d, 1H, *J*= 1.9 Hz), 7.05 (s,br, 1H), 7.01 (dd, 1H, *J*= 8.6, 2.0 Hz), 6.81 (d, 1H, *J*= 8.7 Hz), 6.70 (d, 1H, *J*= 8.6 Hz), 6.67 (d, 1H, *J*= 2.5 Hz), 6.52 (dd, 1H, *J*= 8.7, 2.6 Hz), 4.25-4.22 (m, 4H), 4.19-4.17 (m, 4H); ^{13}C-NMR (100 MHz, $CDCl_3$): δ 163.9 (C), 143.7 (C), 143.756 (C), 142.734 (C), 141.0 (C), 139.4 (C), 135.9 (C), 132.1 (CH), 131.0 (C), 130.2 (CH), 129.5 (CH), 129.1 (C), 125.3 (CH), 117.6 (CH), 117.3 (CH), 117.1 (CH), 114.3 (CH), 113.2 (CH), 110.4 (CH), 64.4 (CH_2), 64.3 (CH_2), 64.2 (CH_2), 64.17 (CH_2); IR (neat): *v*= 3237 (s), 2932 (s), 1601 (m), 1502 (s), 1300 (m), 1281 (m), 1240 (m), 1204 (m), 1152 (s), 1063 (s), 966 (m), 920 (m), 885 (s), 808.17 (m), 681 (s), 621 (m), 580 (s), 548 (s) cm^{-1}; MS: *m/z*= 469.0 $[M+H]^+$.

Compound **3eg**

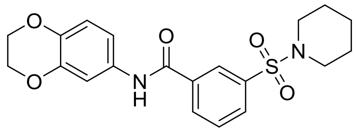

White solid (recrystallization in methanol; 93 mg, 77%); m.p.= 186 - 187 °C; ^{1}H-NMR (400 MHz, DMSO-d_6): δ 10.35 (s, 1H), 8.27-8.23 (m, 2H), 7.93-7.91 (m, 1H), 7.80 (dd, 1H, *J*= 7.8 Hz), 7.38 (d, 1H, *J*= 2.4 Hz), 7.19 (dd, 1H, *J*= 8.7, 2.4 Hz), 6.85 (d, 1H, *J*= 8.7 Hz), 4.27-4.22 (m, 4H); ^{13}C-NMR (100 MHz, DMSO-d_6): δ 164.0 (C),

143.3 (C), 140.4 (C), 136.5 (C), 136.4 (C), 132.8 (C), 132.4 (CH), 130.6 (CH), 126.7 (CH), 117.2 (CH), 114.4 (CH), 110.2 (CH), 64.6 (CH_2), 64.4 (CH_2), 47.1 (CH_2), 25.1 (CH_2), 23.4 (CH_2); IR (neat): *v*= 3273 (w), 2941 (w), 2853 (w), 1643 (m), 1611 (m), 1506 (s), 1429 (m), 1416 (m), 1337 (s), 1300 (s), 1281 (s), 1242 (m), 1204 (m), 1180 (s), 1069 (s), 932 (s), 862 (m), 737 (m), 710 (s), 596 (s), 579 (s), 501 (m) cm^{-1}; MS: *m/z*= 403.0 $[M+H]^+$.

Compound **1**

^{1}H-NMR

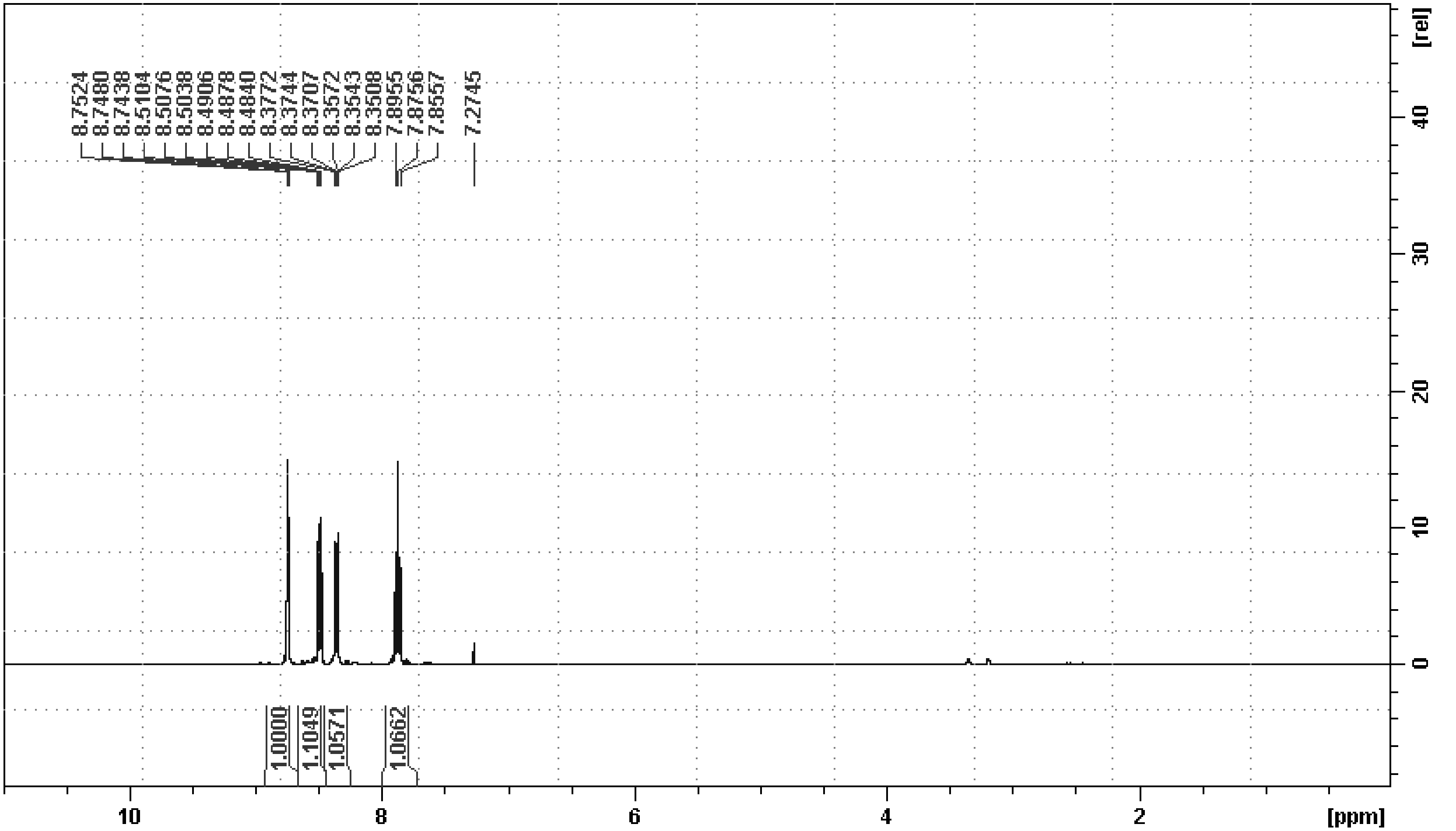

^{13}C-NMR

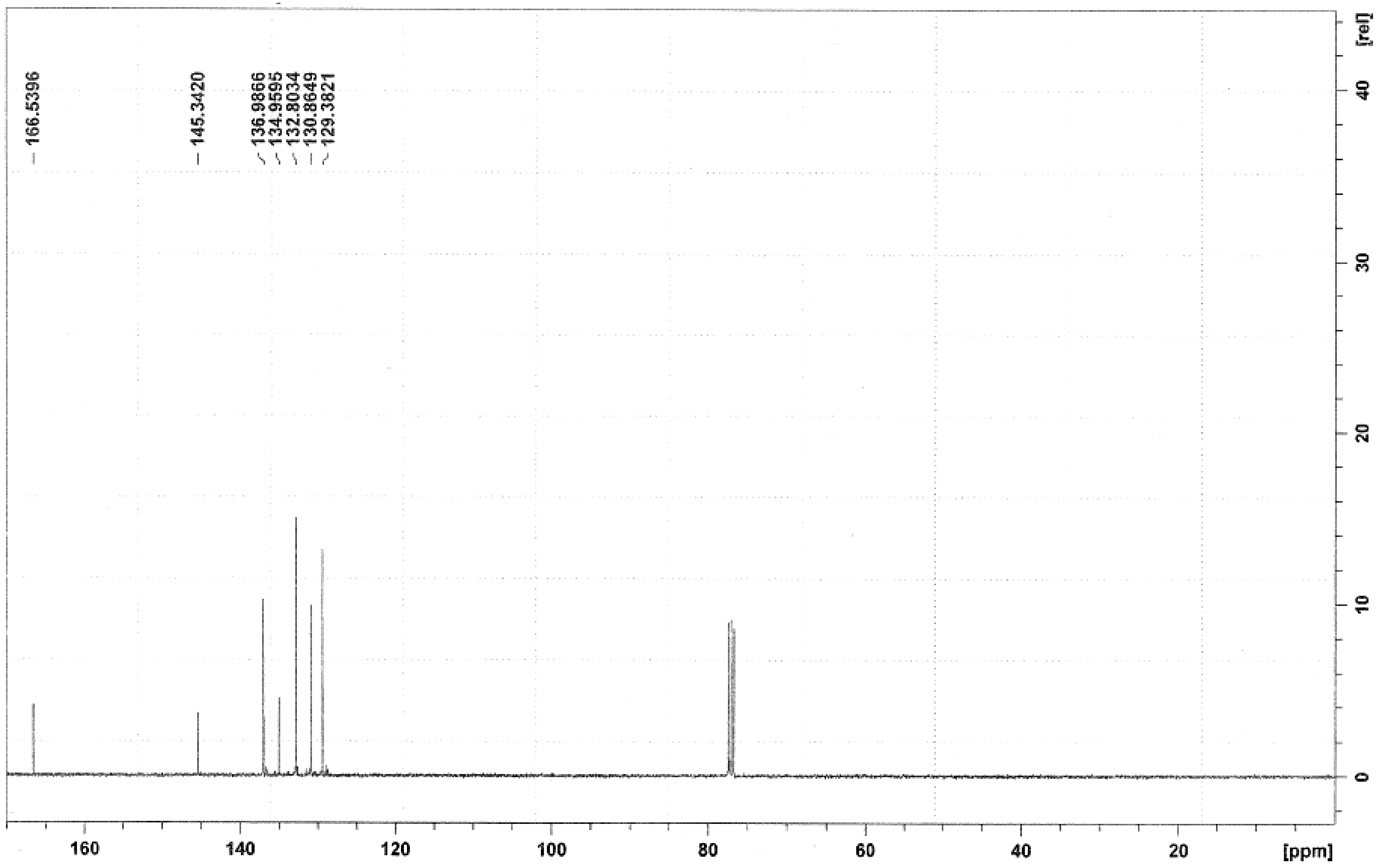

166.5396
145.3420
136.9866
134.9595
132.8034
130.8649
129.3821
[rel]
40
30
20
10
0
160
140
120
100
80
60
40
20
[ppm]

Compound **3aa**

^{1}H-NMR

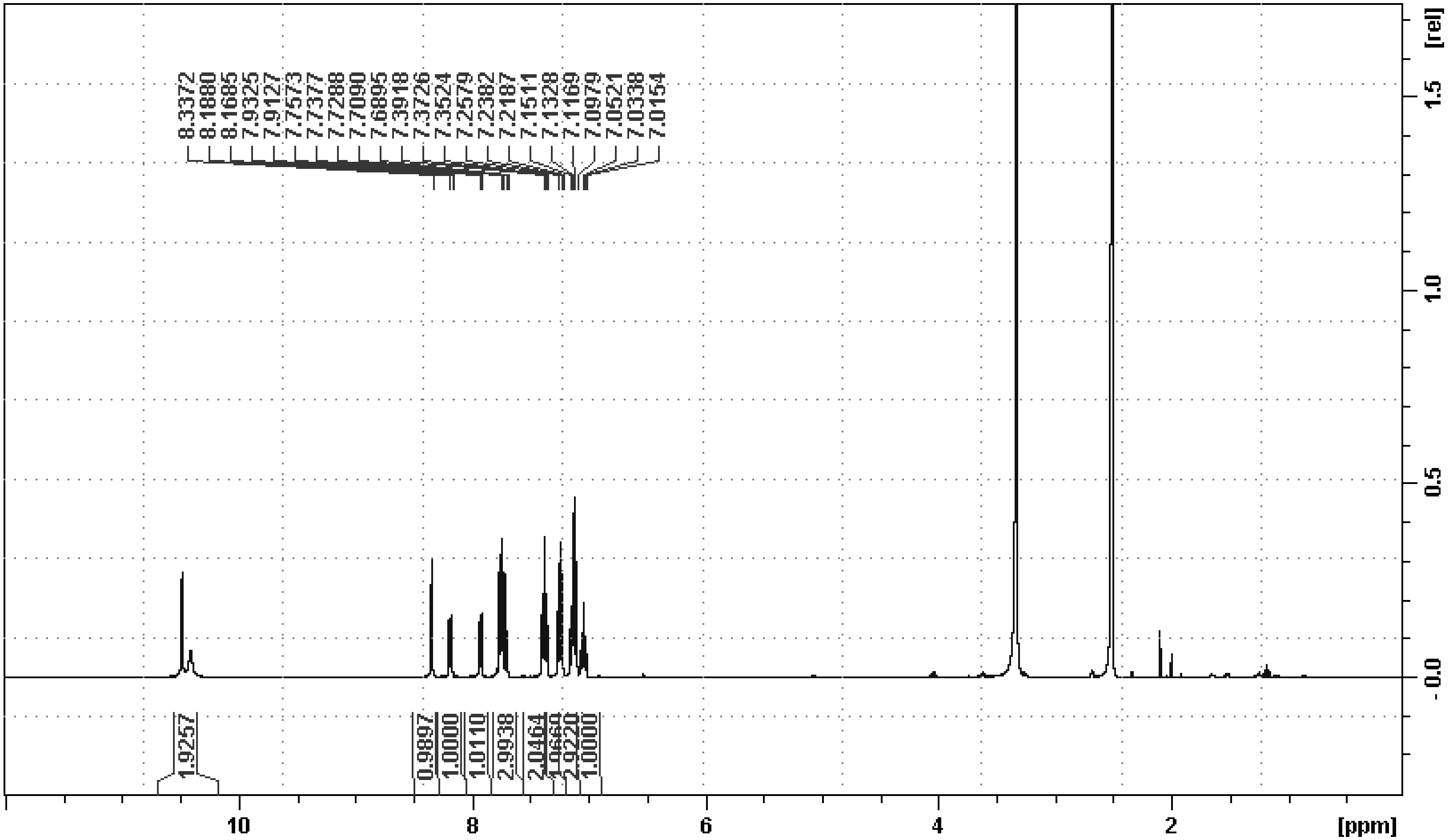

^{13}C-NMR

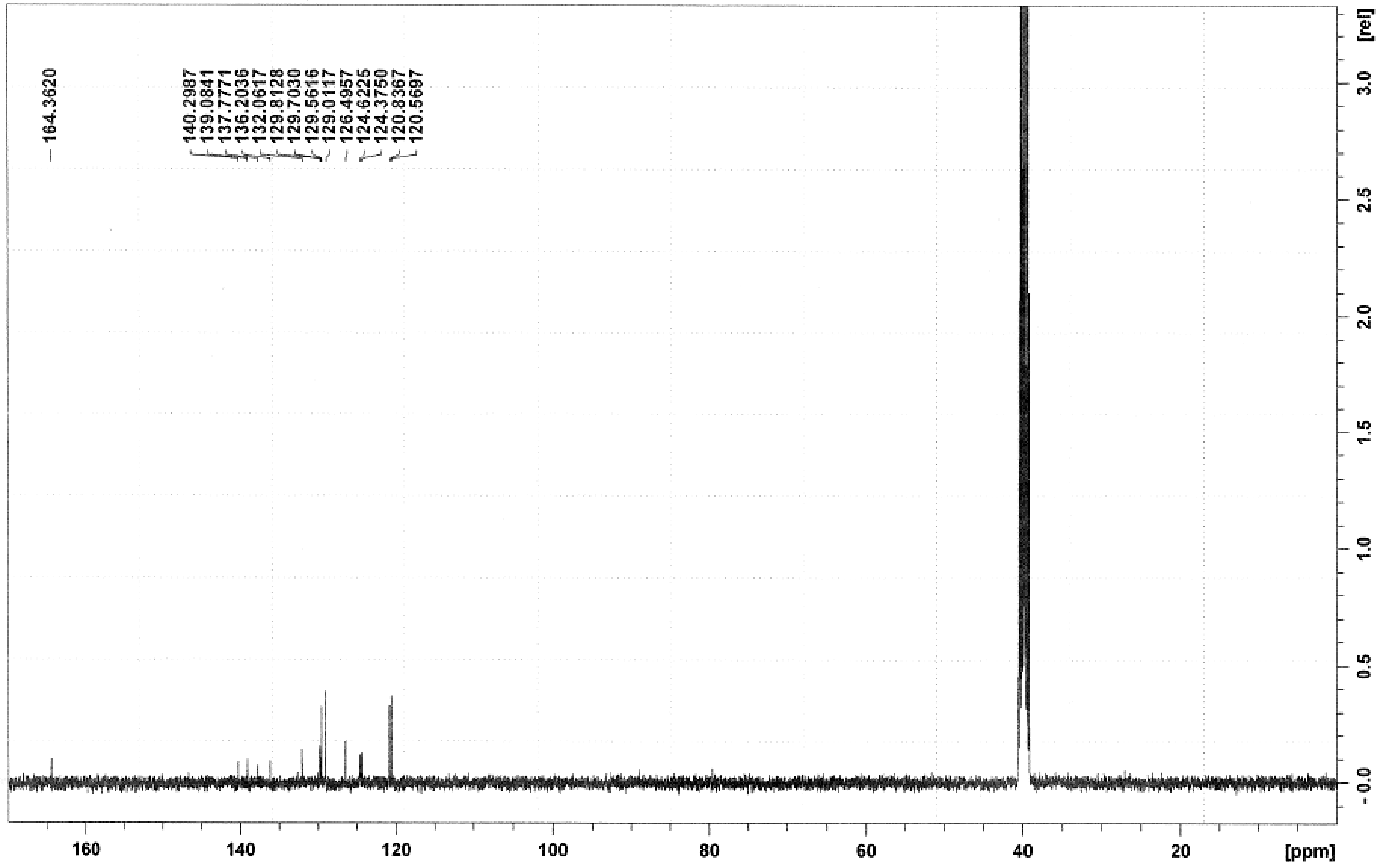

164.3620
140.2987
139.0841
137.7771
136.2036
132.0617
129.8128
129.7030
129.5616
129.0117
126.4957
124.6225
124.3750
120.8367
120.5697
[rel]
3.0
2.5
2.0
1.5
1.0
0.5
-0.0
160
140
120
100
80
60
40
20
[ppm]

Compound **3ab**

^{1}H-NMR

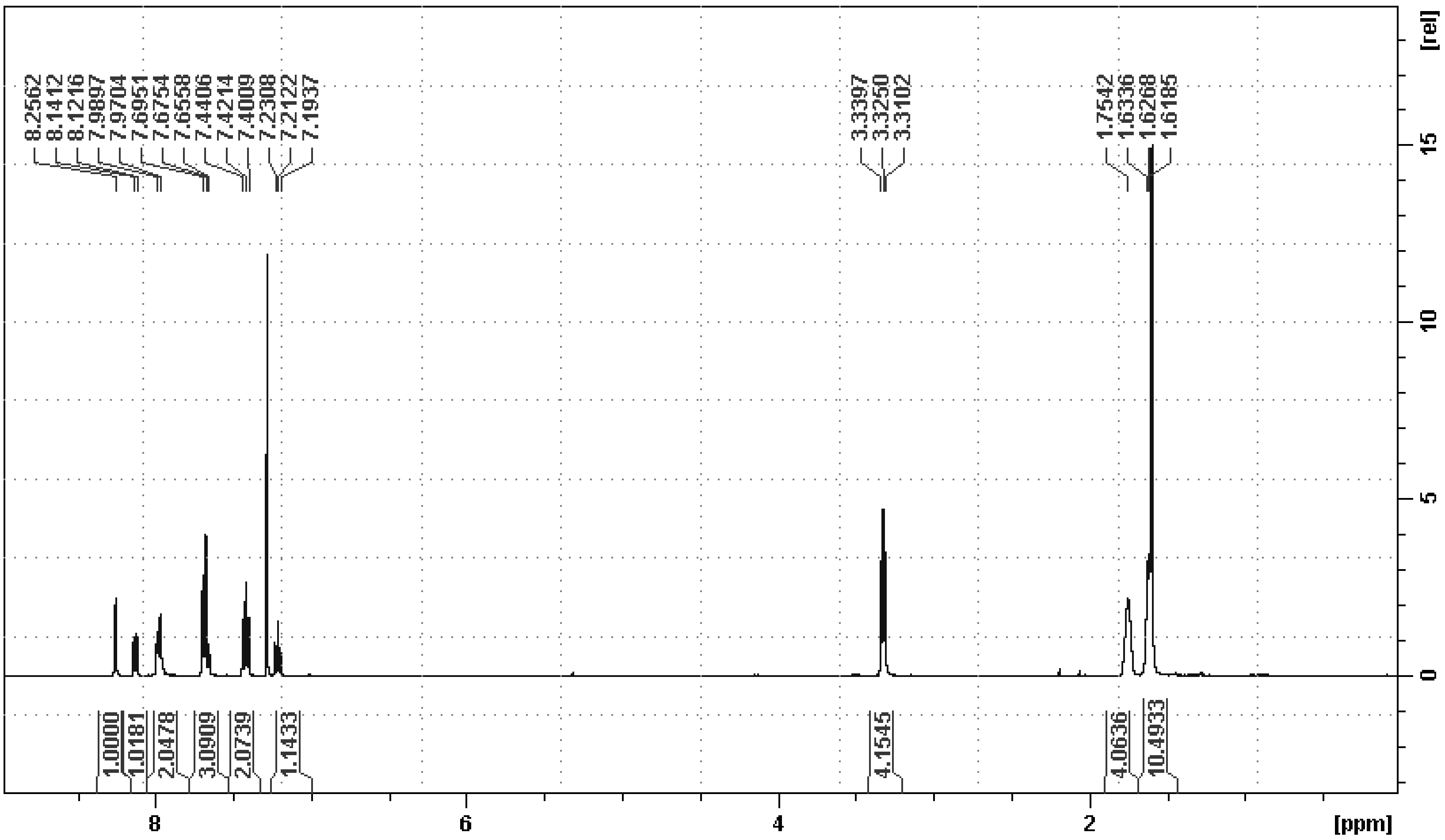

^{13}C-NMR

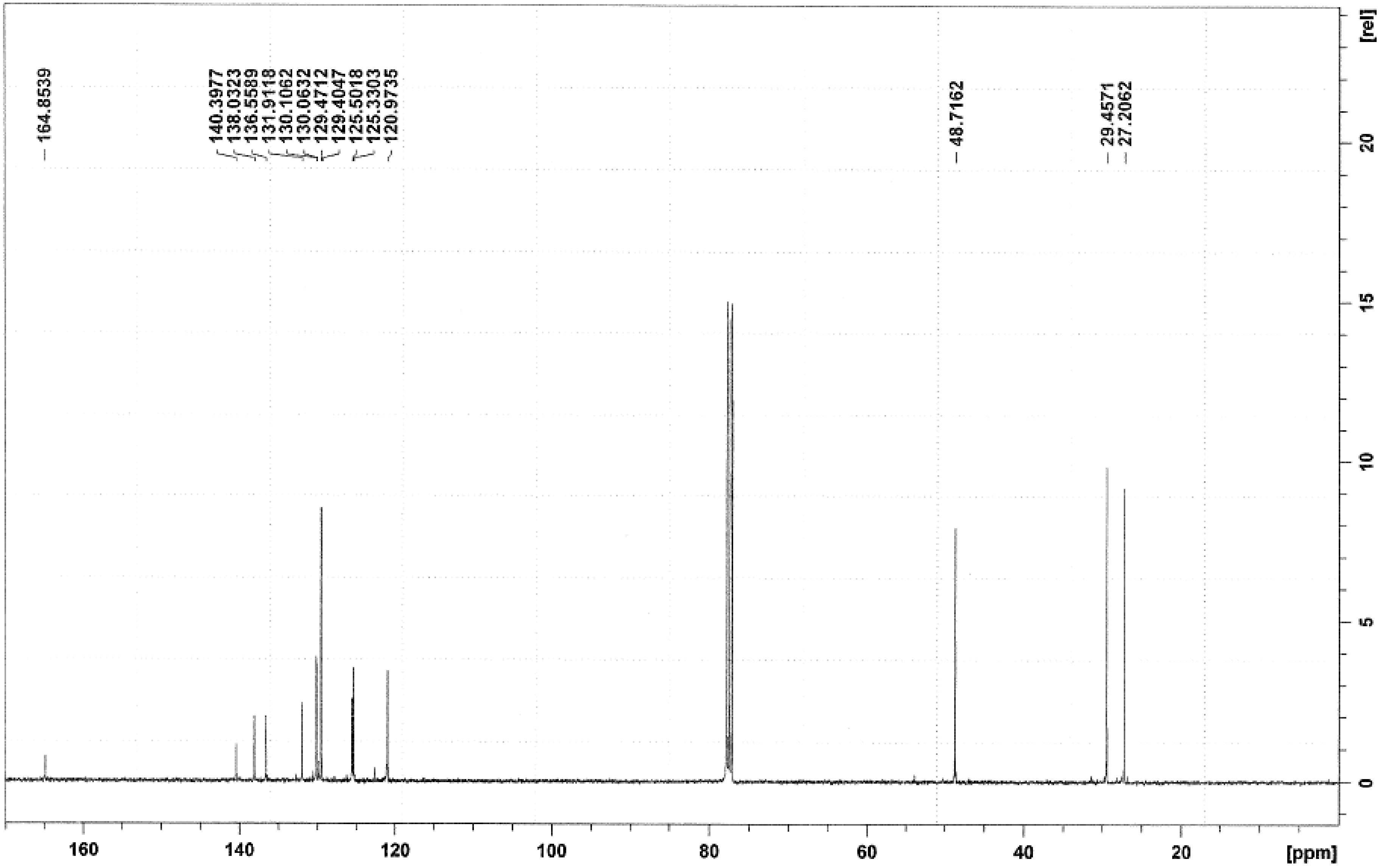

164.8539
140.3977
138.0323
136.5589
131.9118
130.1062
130.0632
129.4712
129.4047
125.5018
125.3303
120.9735
48.7162
29.4571
27.2062
[rel]
[ppm]

Compound **3ac**

^{1}H-NMR

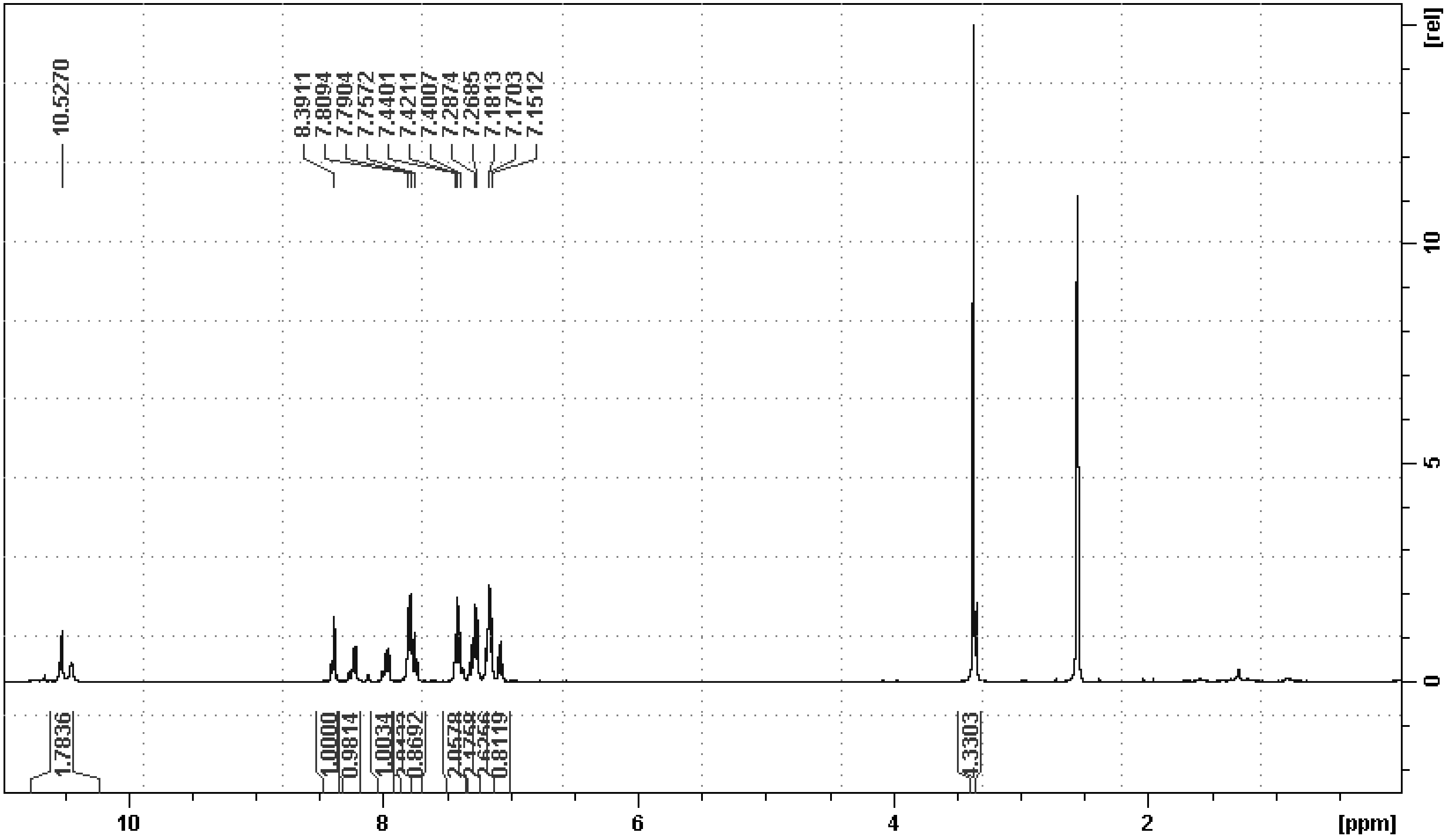

^{13}C-NMR

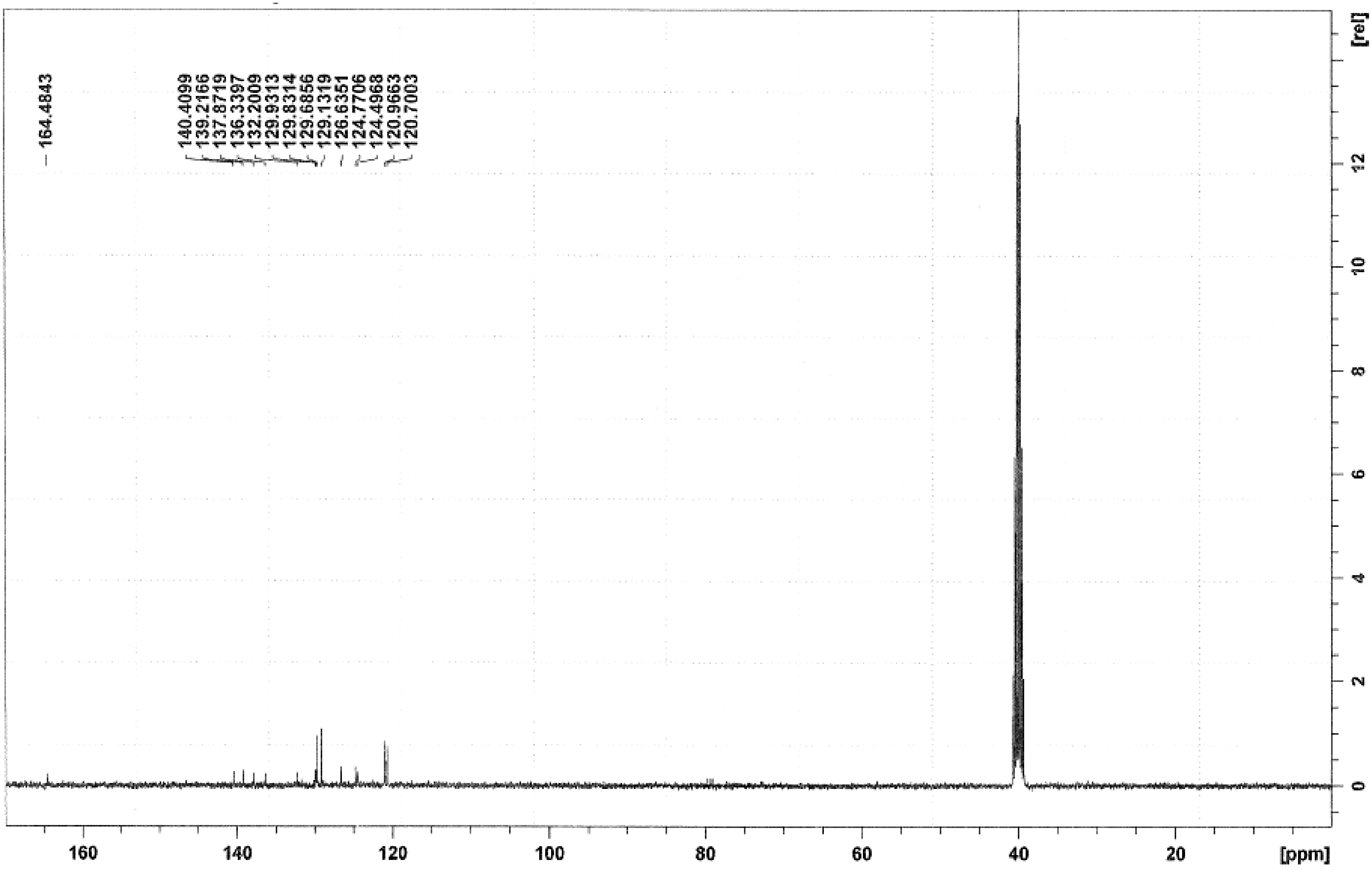
164.4843
140.4099
139.2166
137.8719
136.3397
132.2009
129.9313
129.8314
129.6856
129.1319
126.6351
124.7706
124.4968
120.9663
120.7003
[rel]
[ppm]

Compound **3ae**

^{1}H-NMR

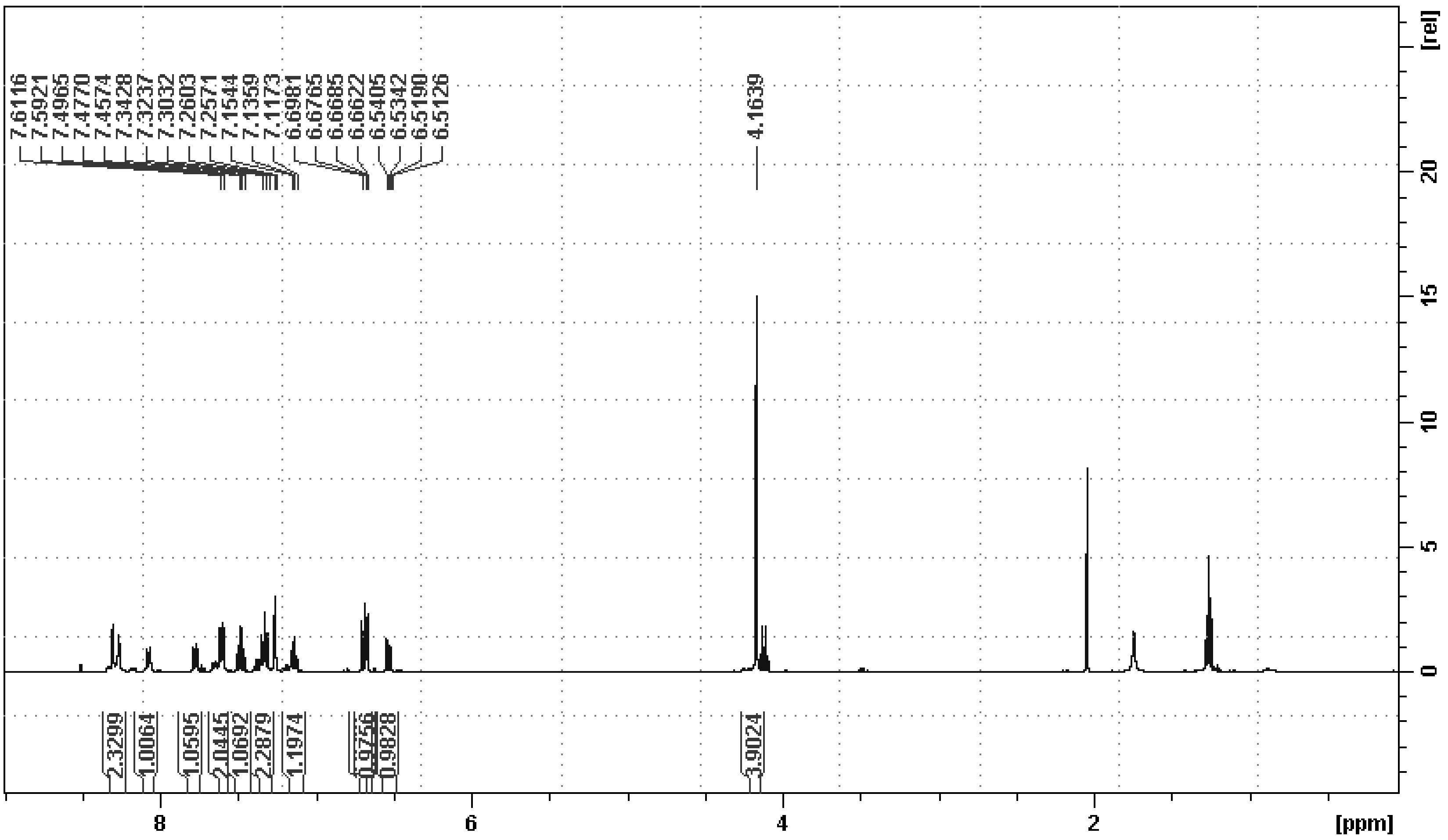

^{13}C-NMR

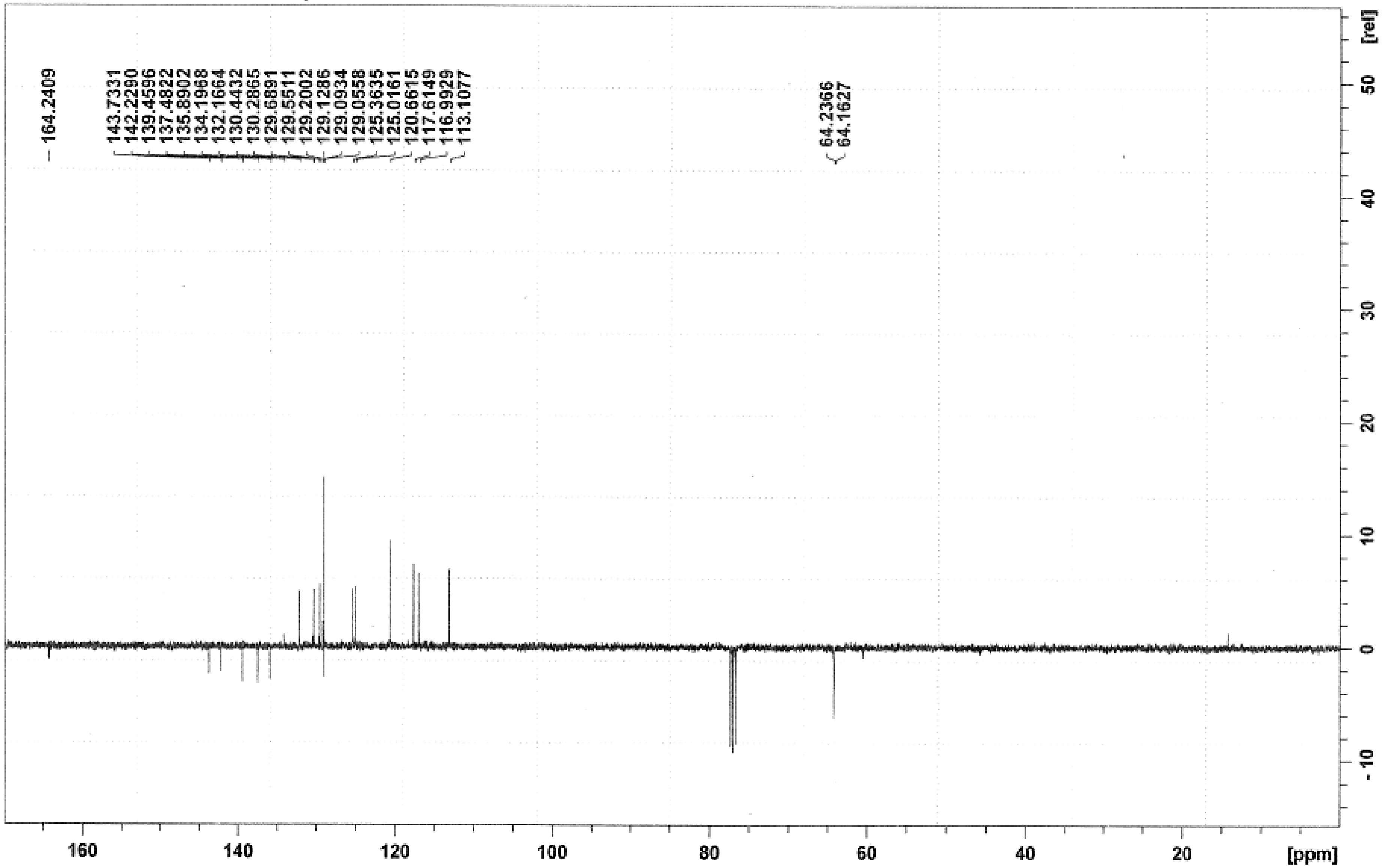

164.2409
143.7331
142.2290
139.4596
137.4822
135.8902
134.1968
132.1664
130.4432
130.2865
129.6891
129.5511
129.2002
129.1286
129.0934
129.0558
125.3635
125.0161
120.6615
117.6149
116.9929
113.1077
64.2366
64.1627
[rel]
[ppm]

Compound **3ag**

^{1}H-NMR

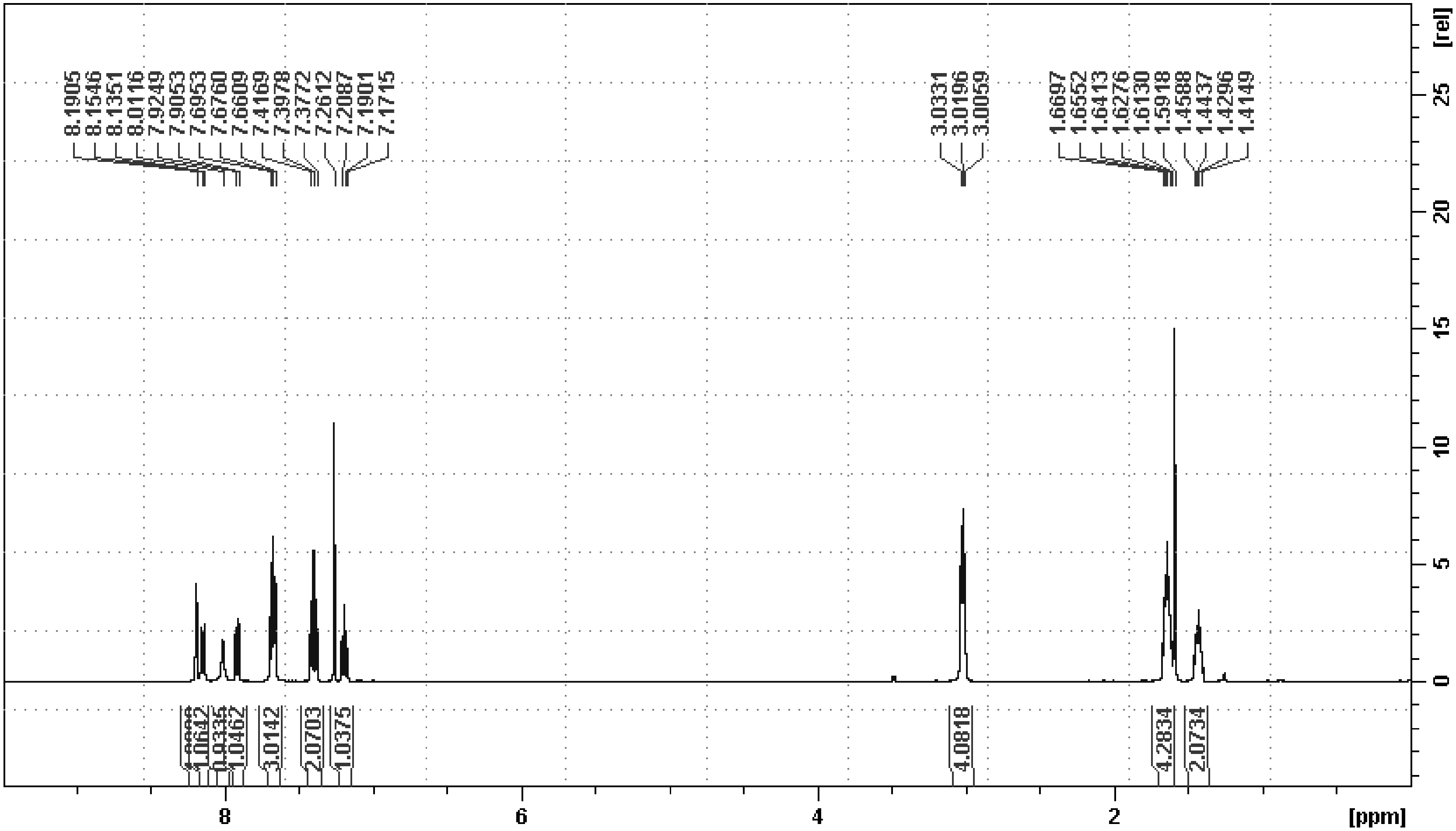

^{13}C-NMR

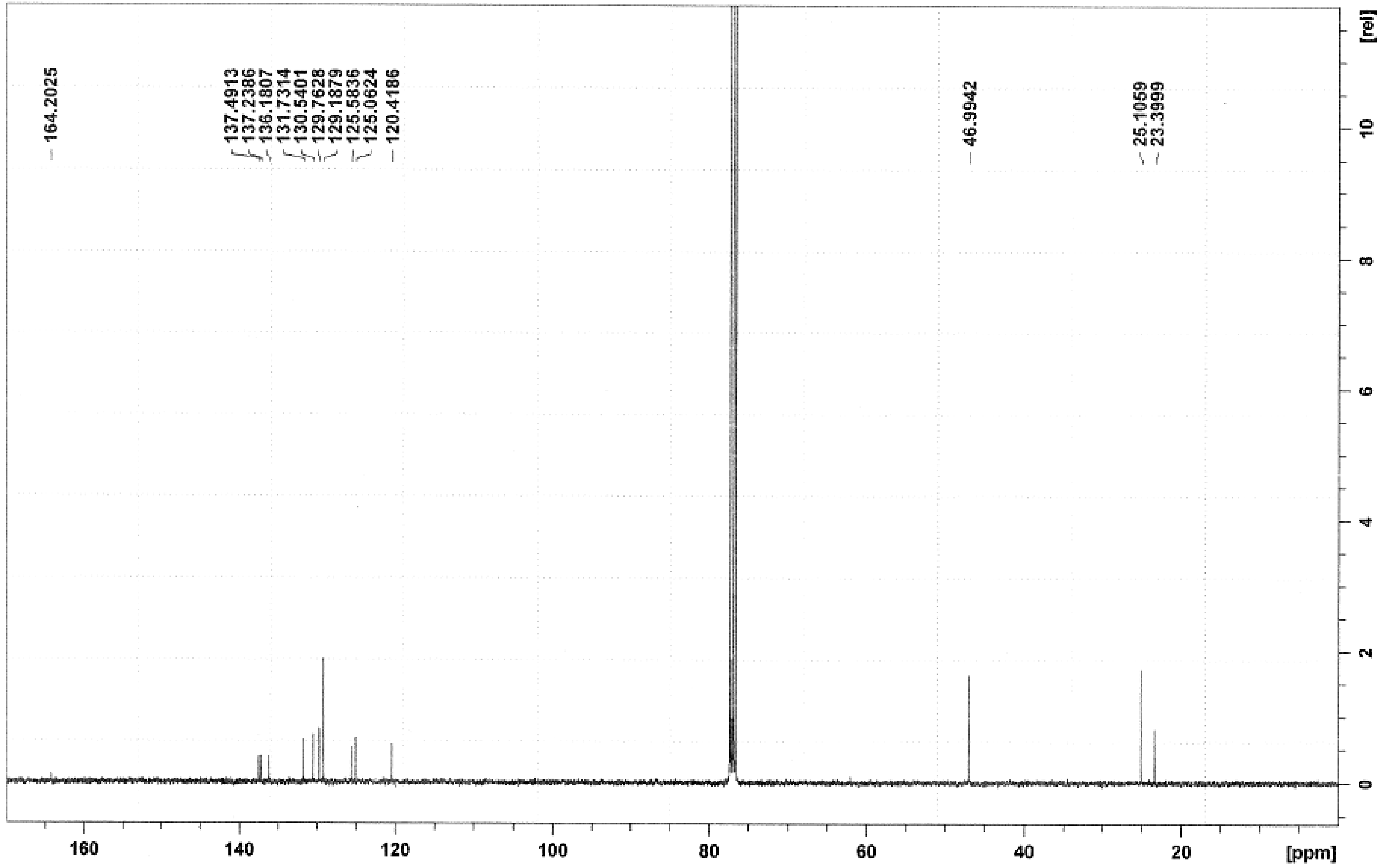

164.2025
137.4913
137.2386
136.1807
131.7314
130.5401
129.7628
129.1879
125.5836
125.0624
120.4186
46.9942
25.1059
23.3999
[rel]
[ppm]

Compound **3ca**

^{1}H-NMR

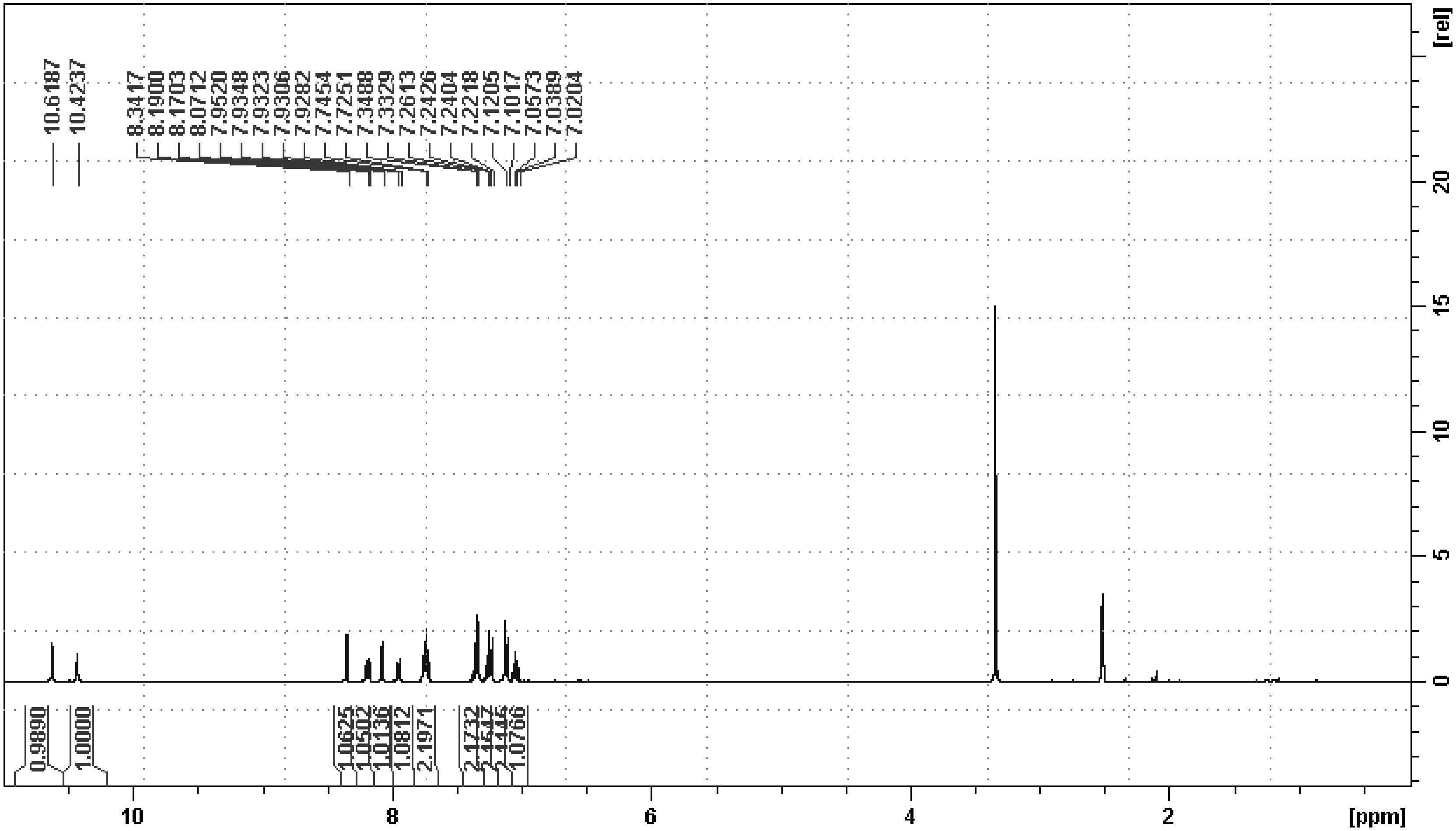

^{13}C-NMR

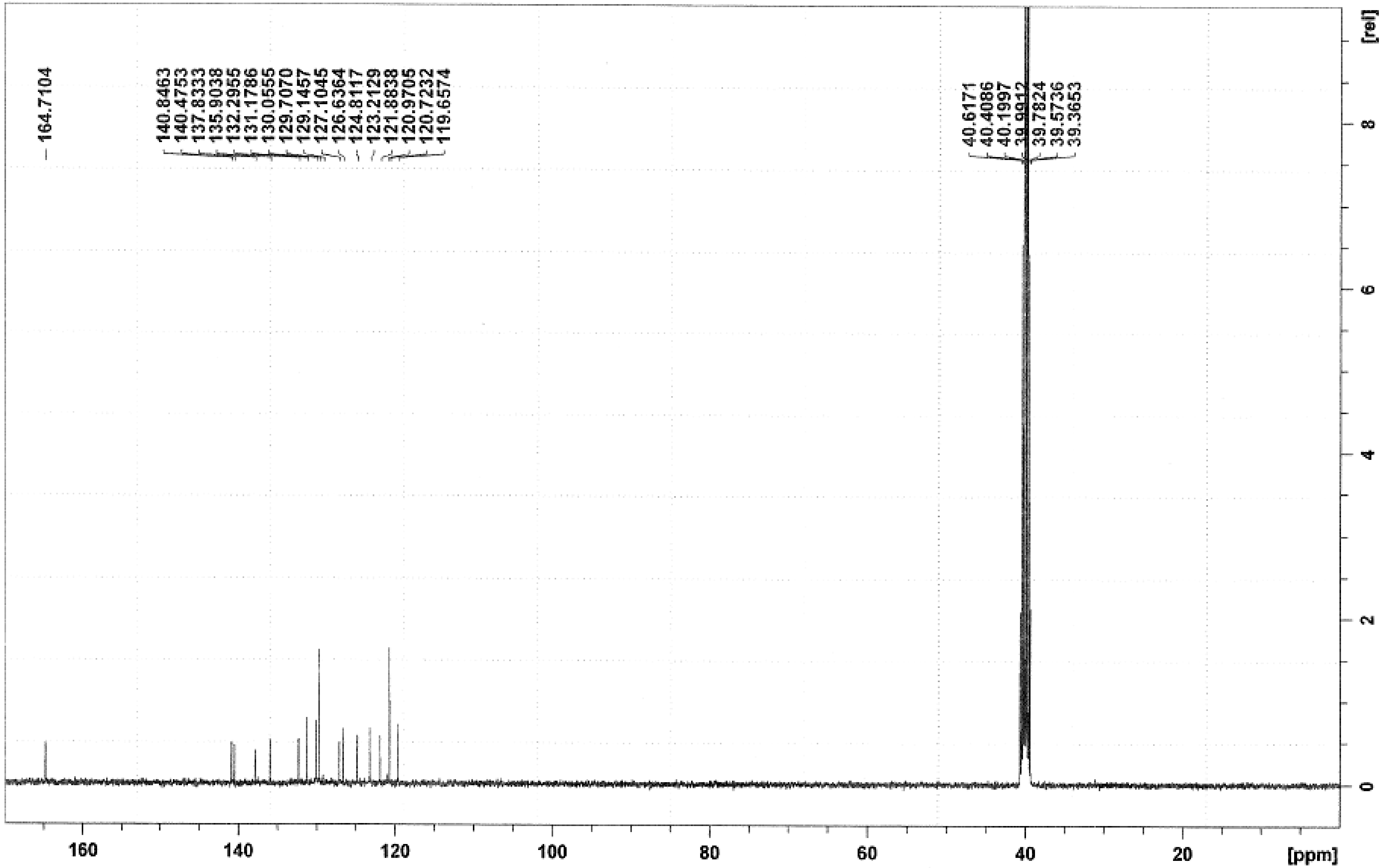

Compound **3cb**

^{1}H-NMR

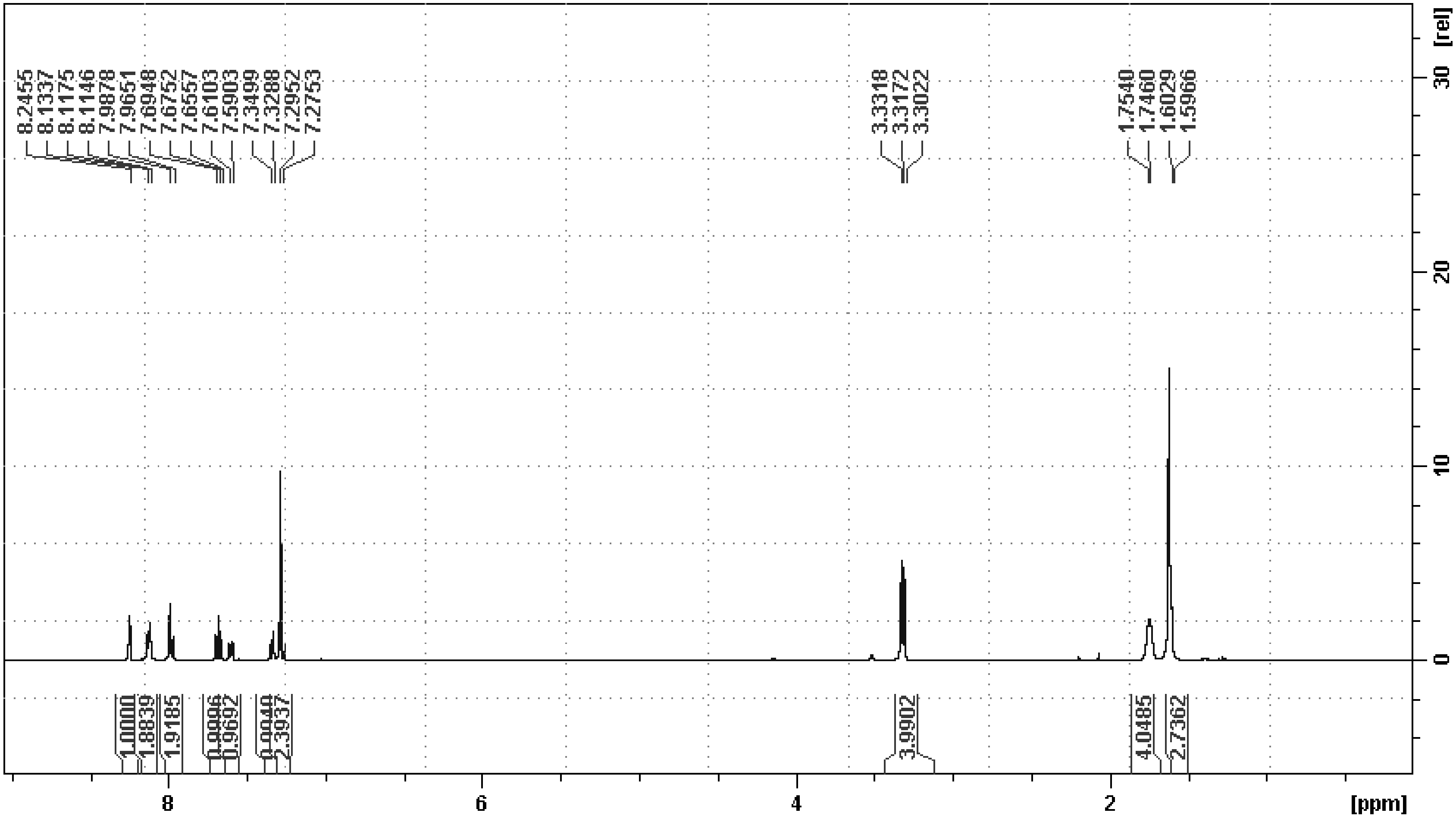

^{13}C-NMR

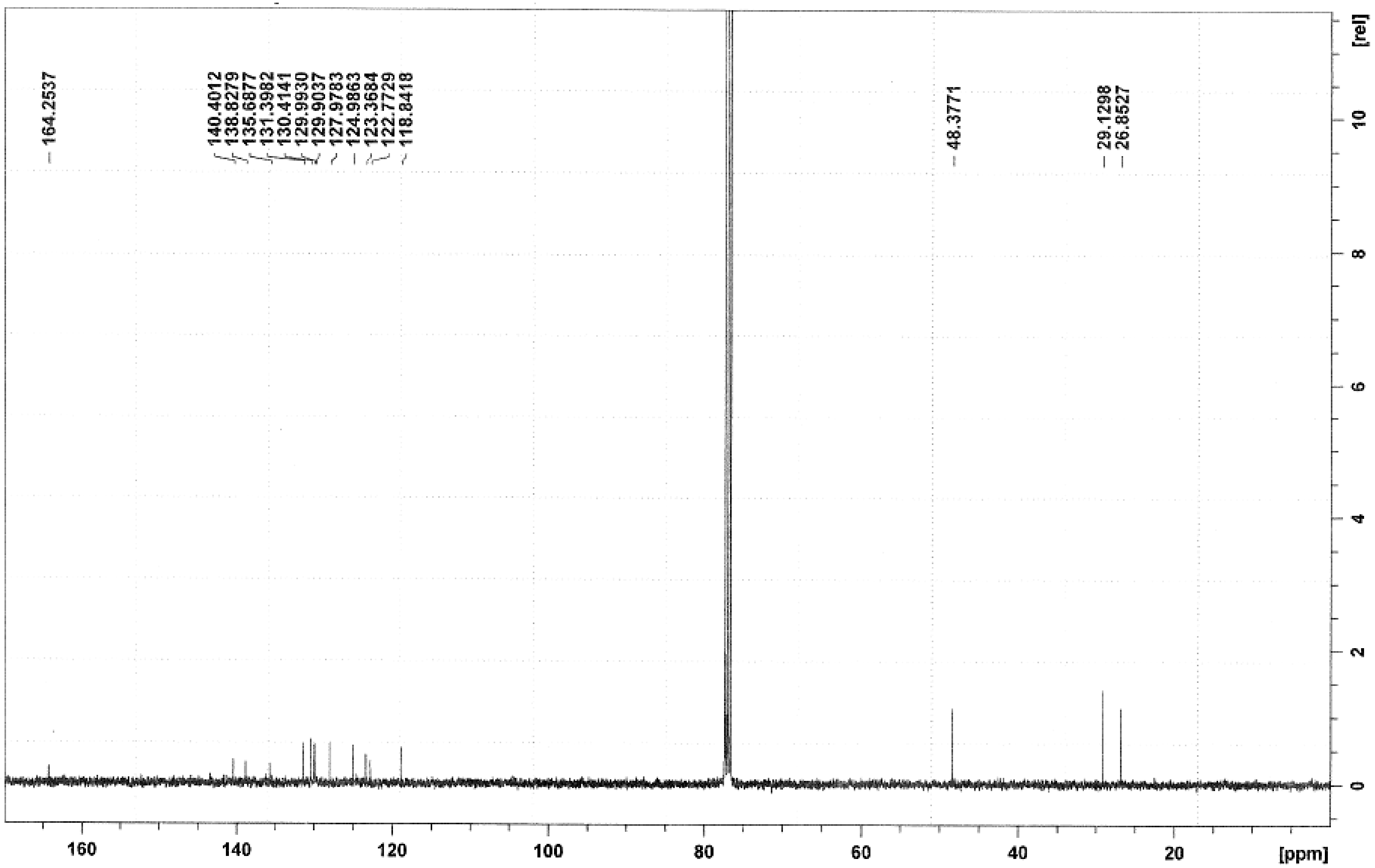
164.2537
140.4012
138.8279
135.6877
131.3982
130.4141
129.9930
129.9037
127.9783
124.9863
123.3684
122.7729
118.8418
48.3771
29.1298
26.8527
[rel]
[ppm]

Compound **3cc**

^{1}H-NMR

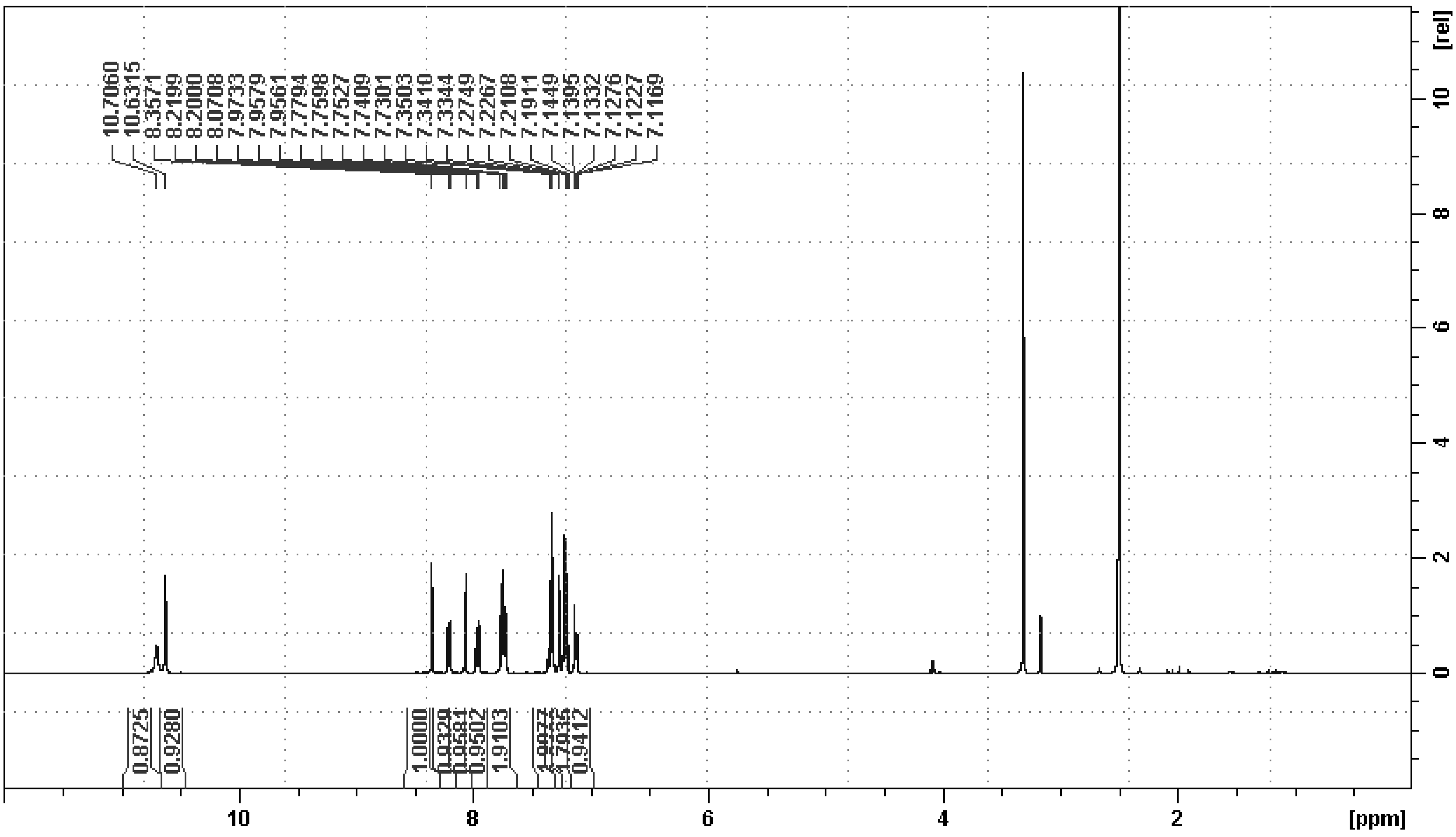

^{13}C-NMR

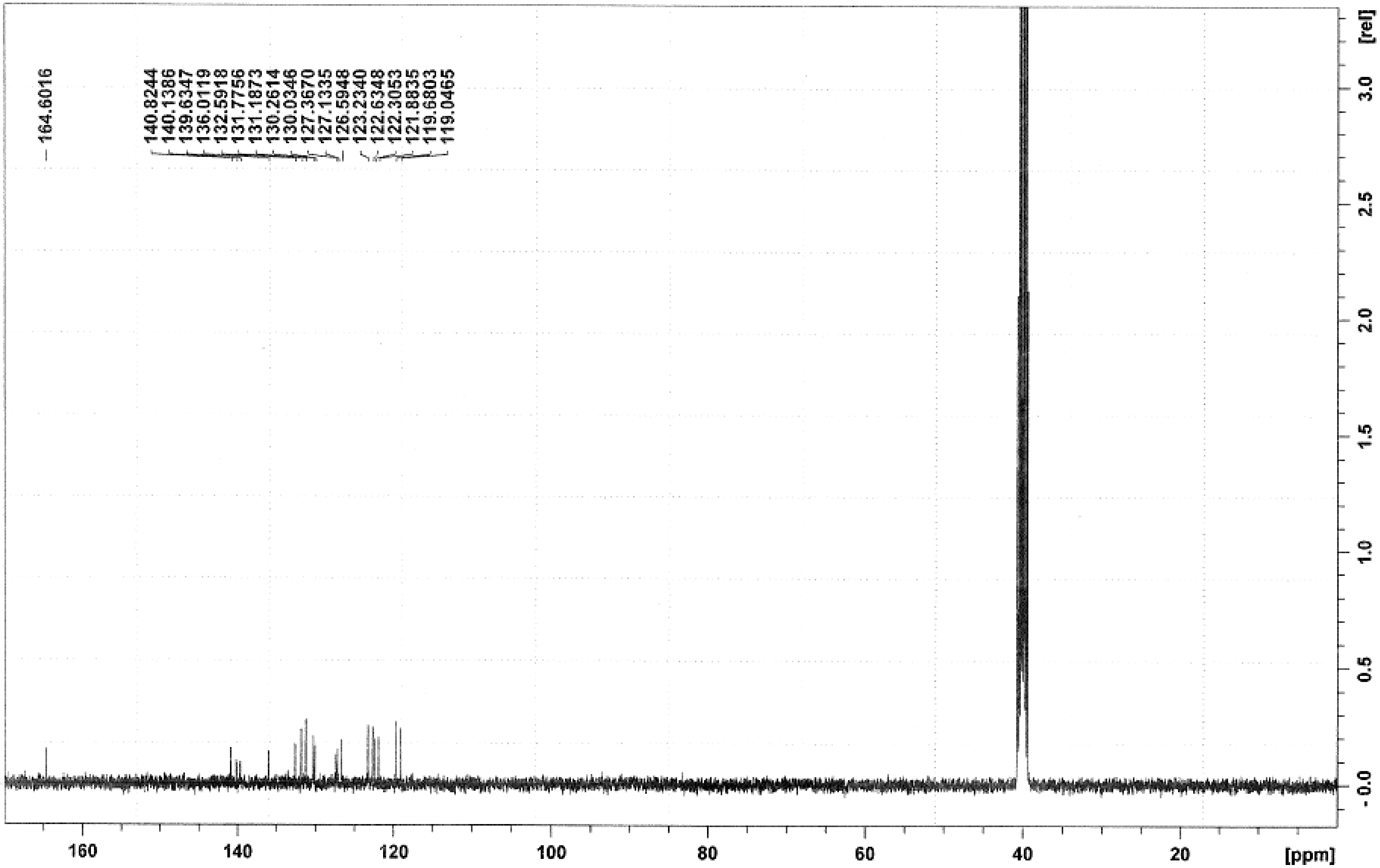

164.6016
140.8244
140.1386
139.6347
136.0119
132.5918
131.7756
131.1873
130.2614
130.0346
127.3670
127.1335
126.5948
123.2340
122.6348
122.3053
121.8835
119.6803
119.0465
[rel]
[ppm]

Compound **3ce**

^{1}H-NMR

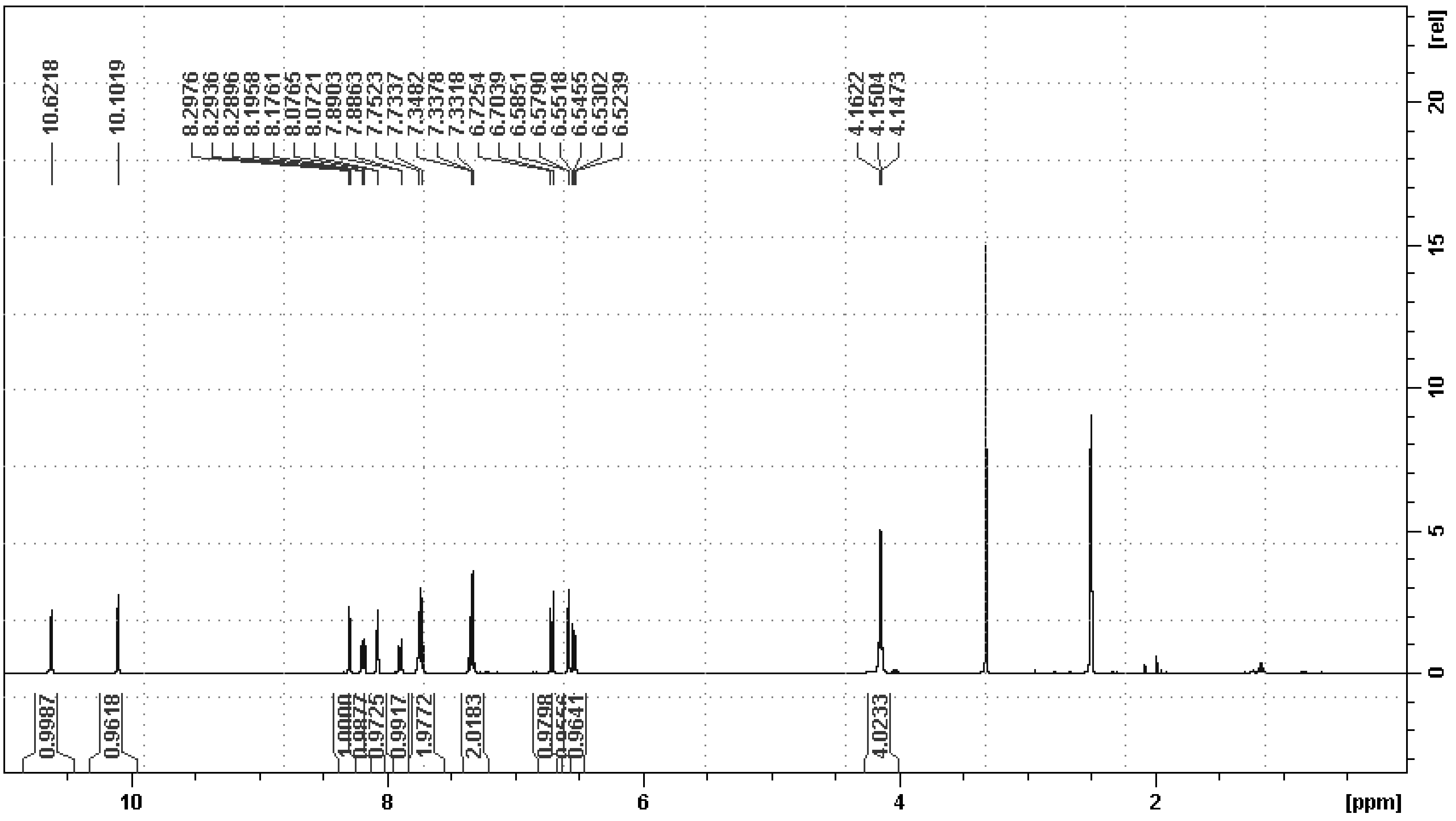

^{13}C-NMR

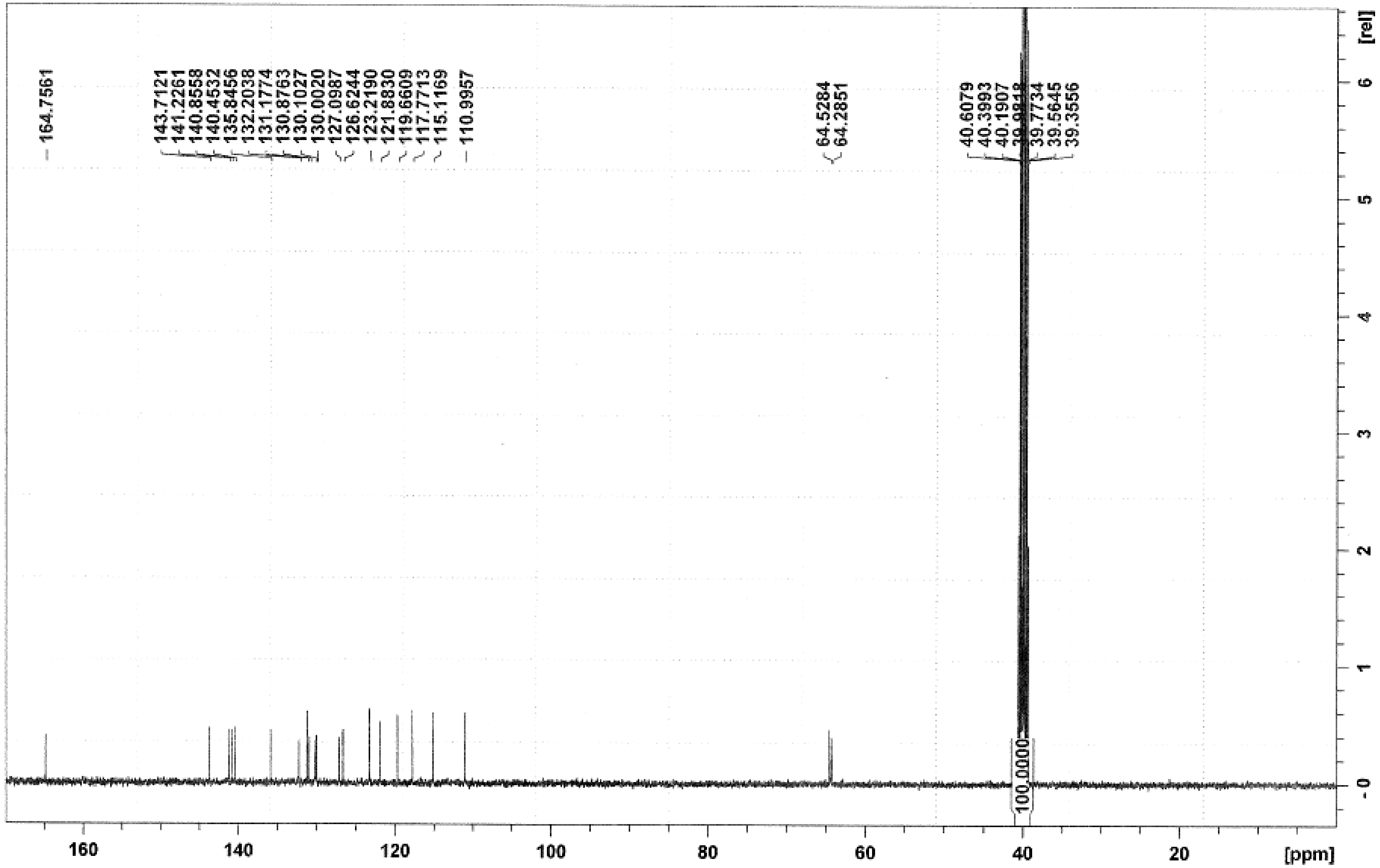

Compound **3cg**

^{1}H-NMR

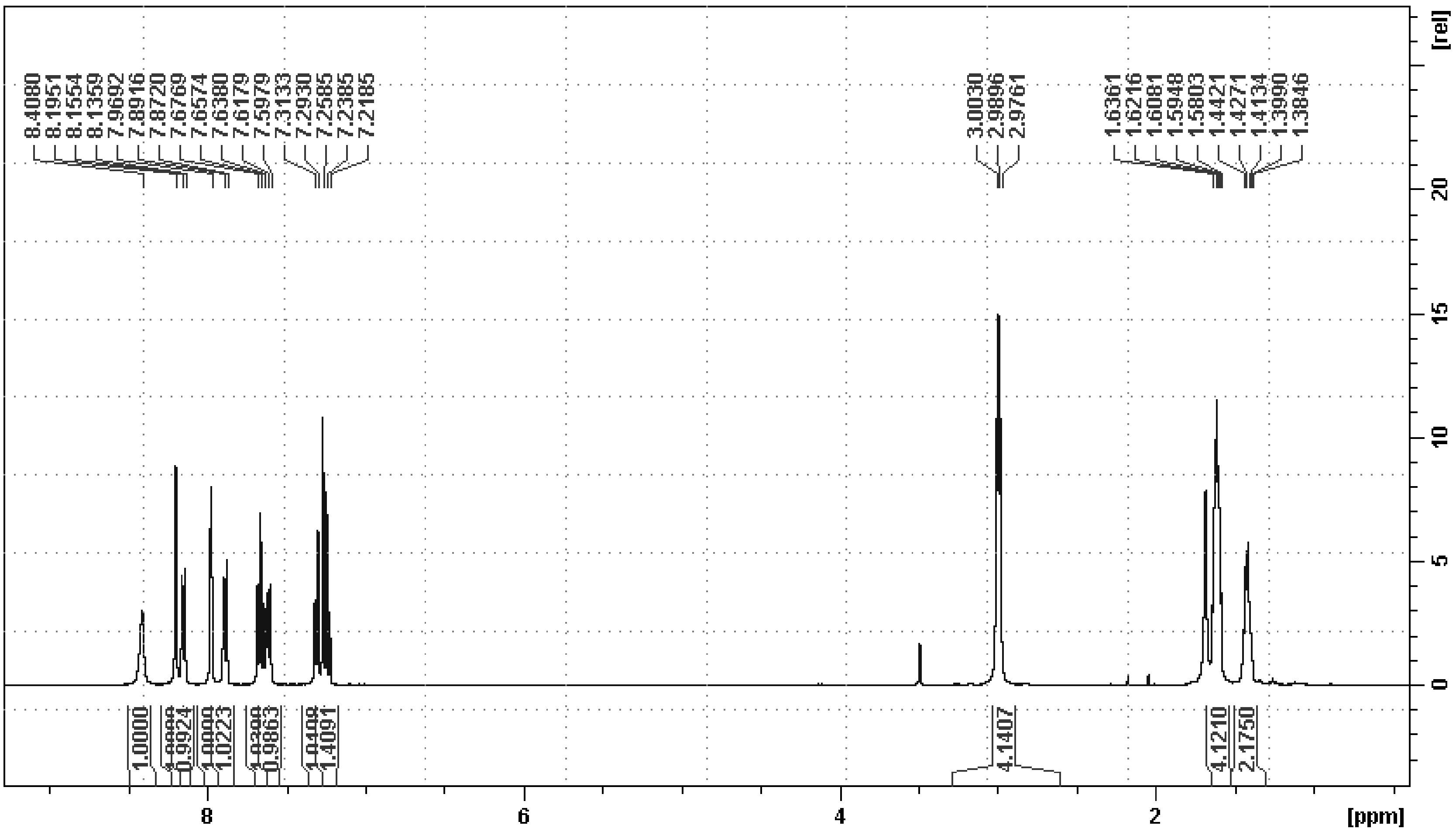

^{13}C-NMR

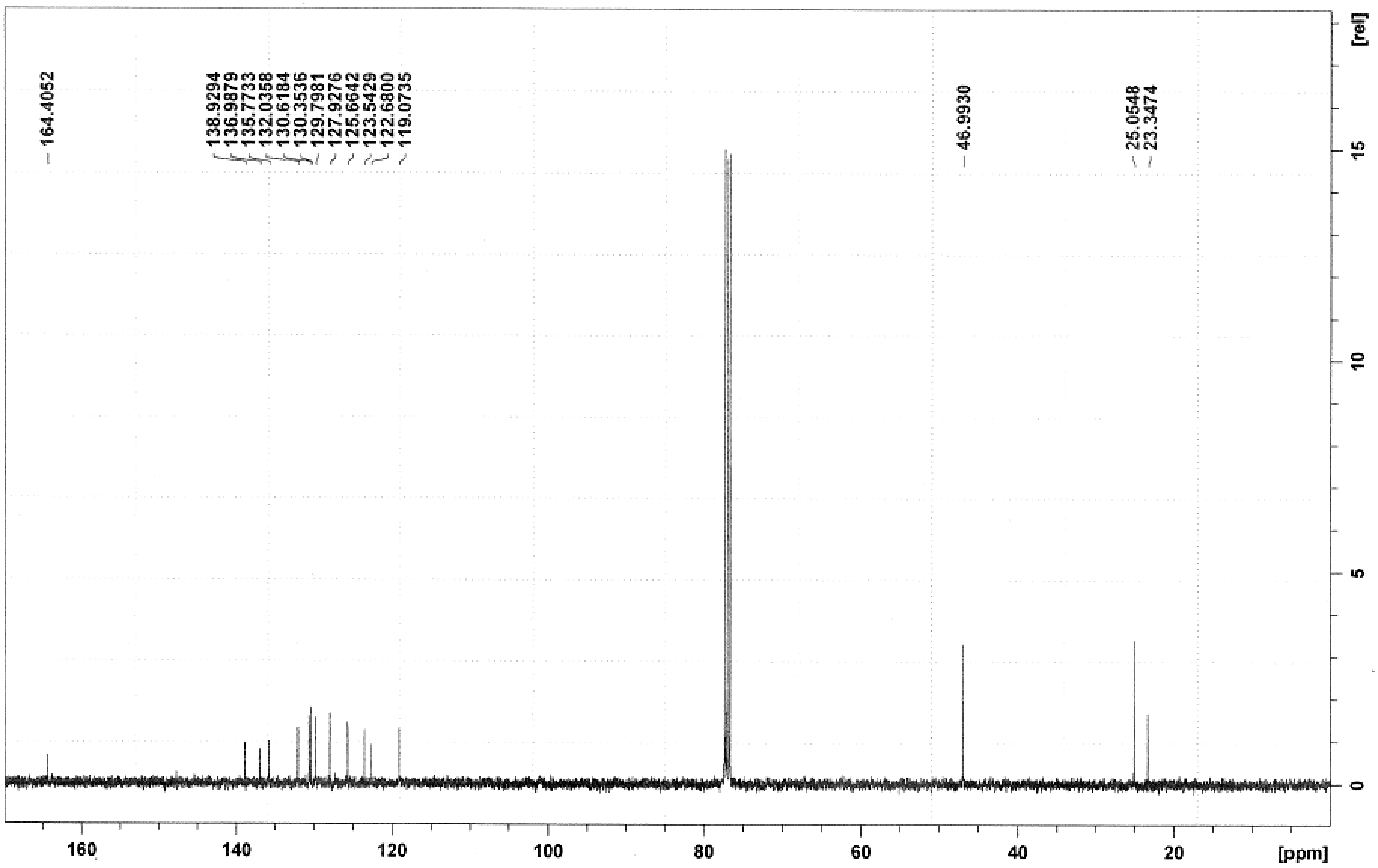
164.4052
138.9294
136.9879
135.7733
132.0358
130.6184
130.3536
129.7981
127.9276
125.6642
123.5429
122.6800
119.0735
46.9930
25.0548
23.3474
[rel]
15
10
5
0
160
140
120
100
80
60
40
20
[ppm]

Compound **3ea**

^{1}H-NMR

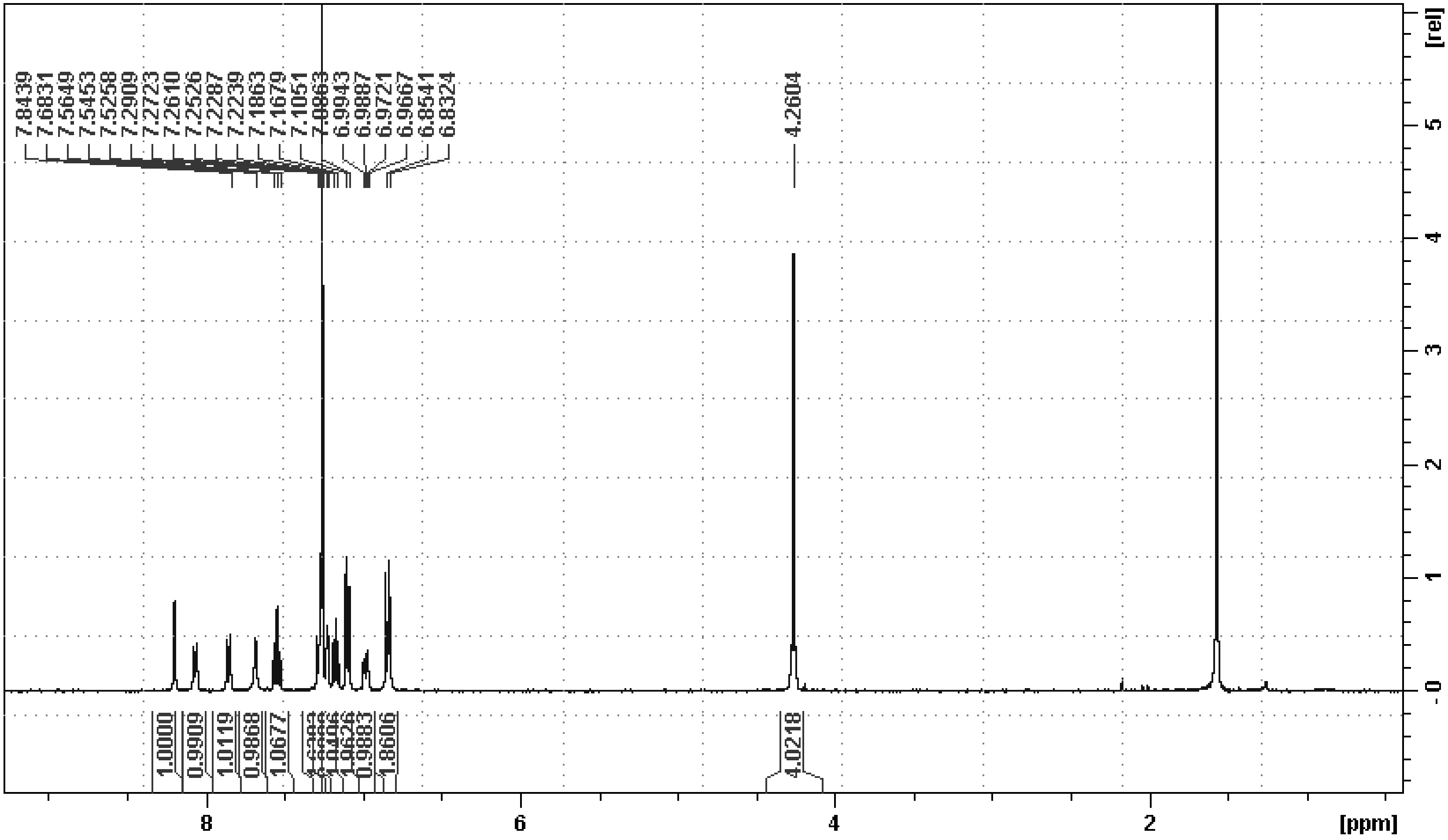

^{13}C-NMR

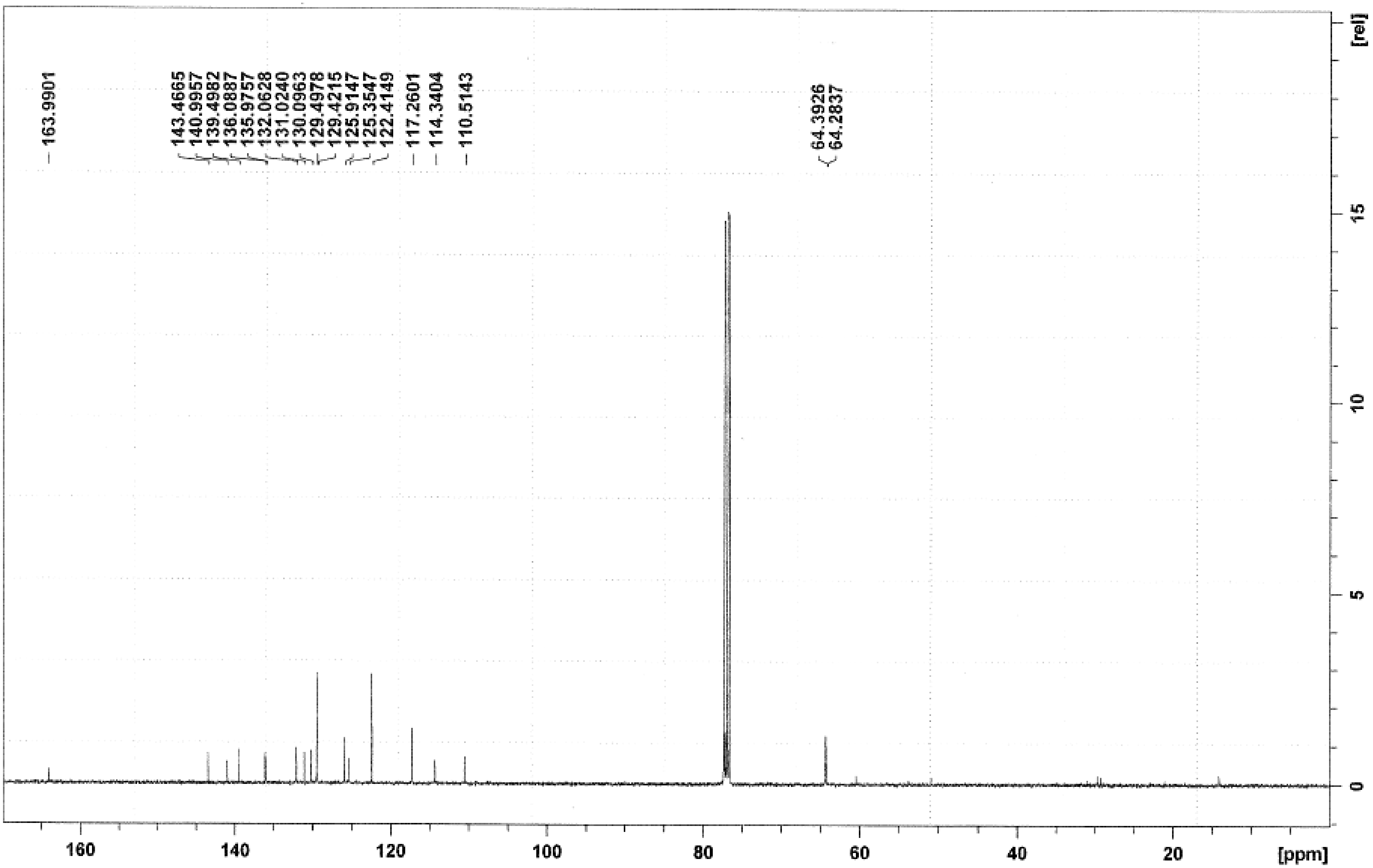
163.9901
143.4665
140.9957
139.4982
136.0887
135.9757
132.0628
131.0240
130.0963
129.4978
129.4215
125.9147
125.3547
122.4149
117.2601
114.3404
110.5143
64.3926
64.2837
[rel]
[ppm]

Compound **3eb**

^{1}H-NMR

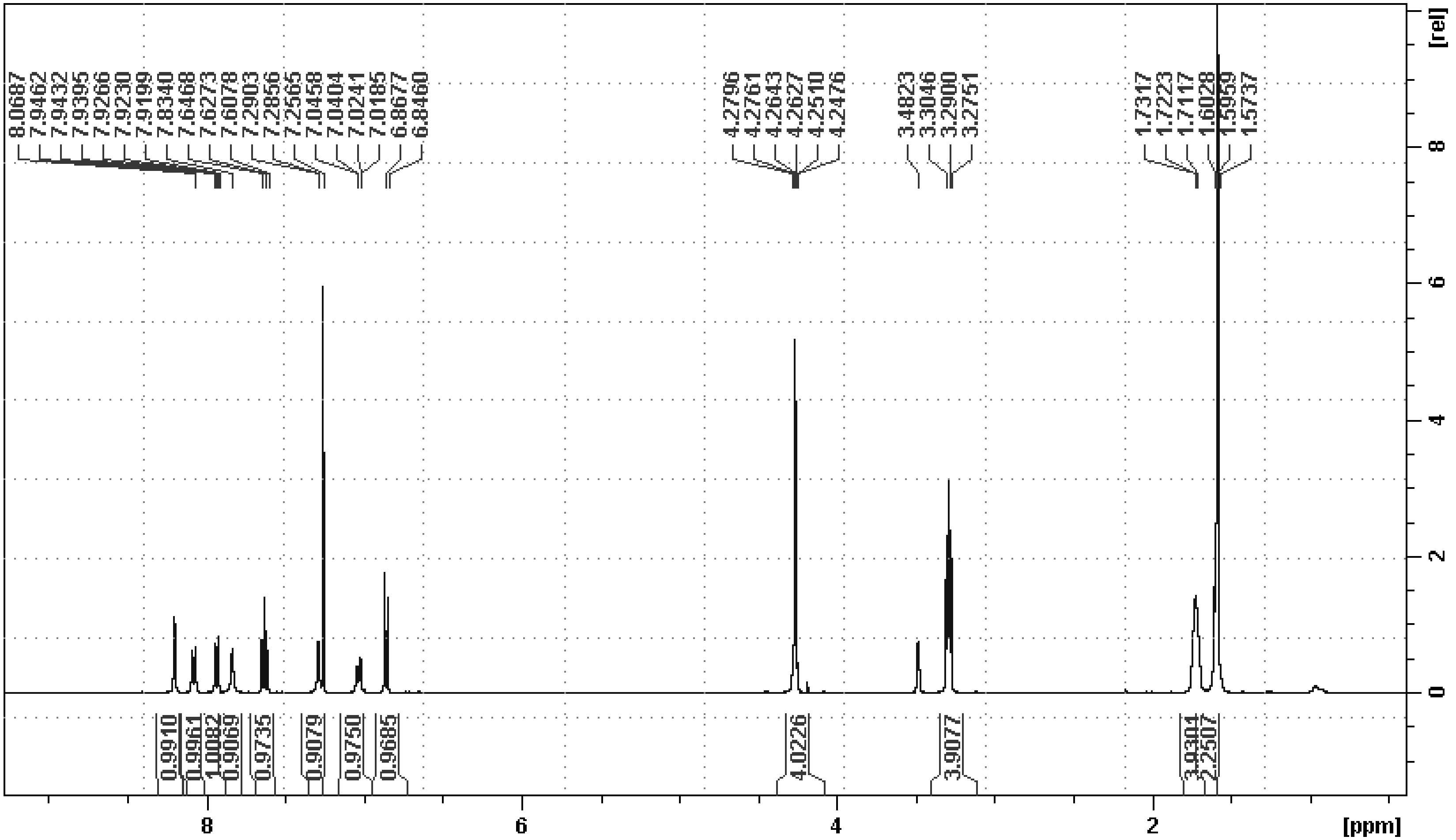

^{13}C-NMR

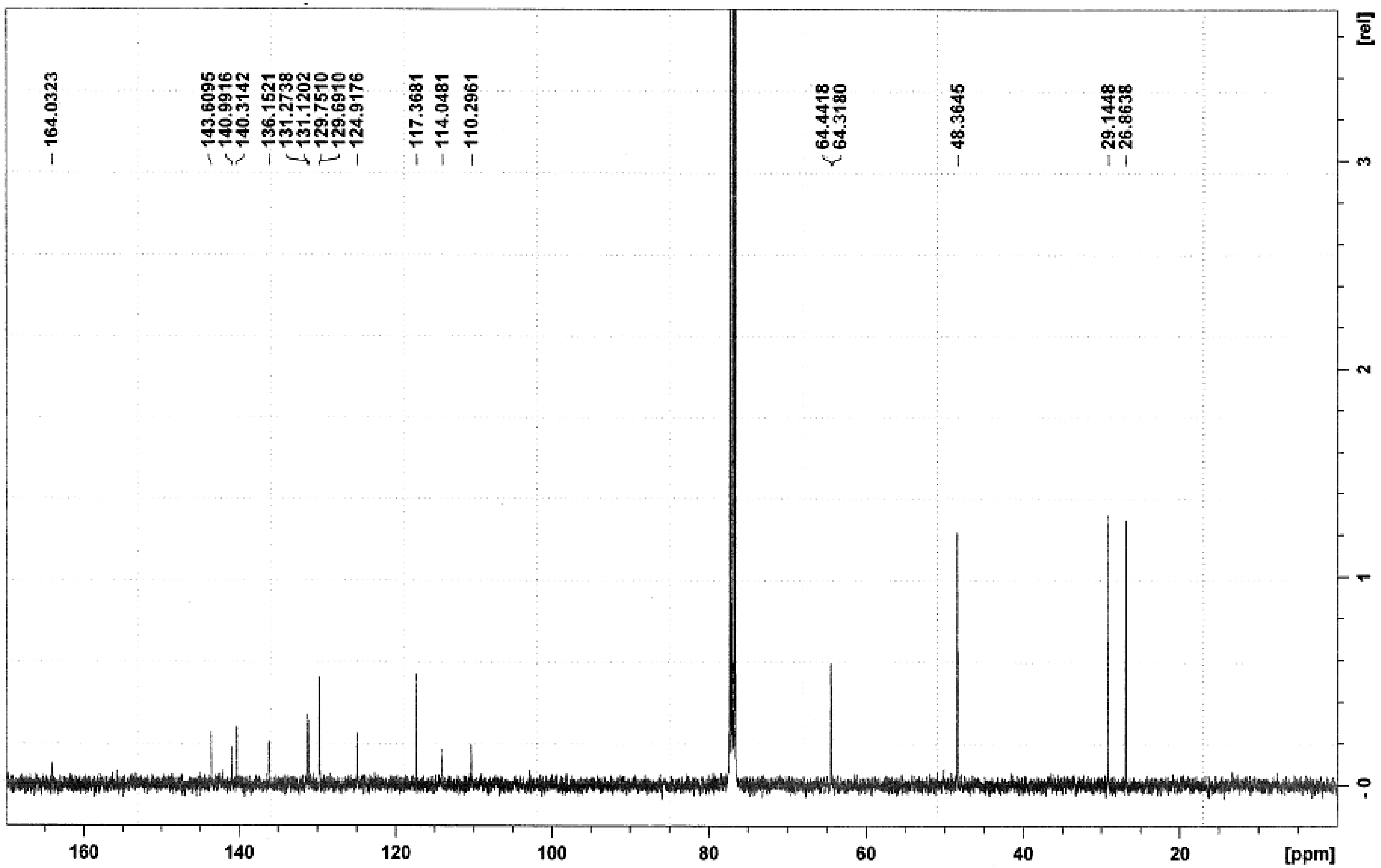
164.0323
143.6095
140.9916
140.3142
136.1521
131.2738
131.1202
129.7510
129.6910
124.9176
117.3681
114.0481
110.2961
64.4418
64.3180
48.3645
29.1448
26.8638
[rel]
[ppm]

Compound **3ec**

^{1}H-NMR

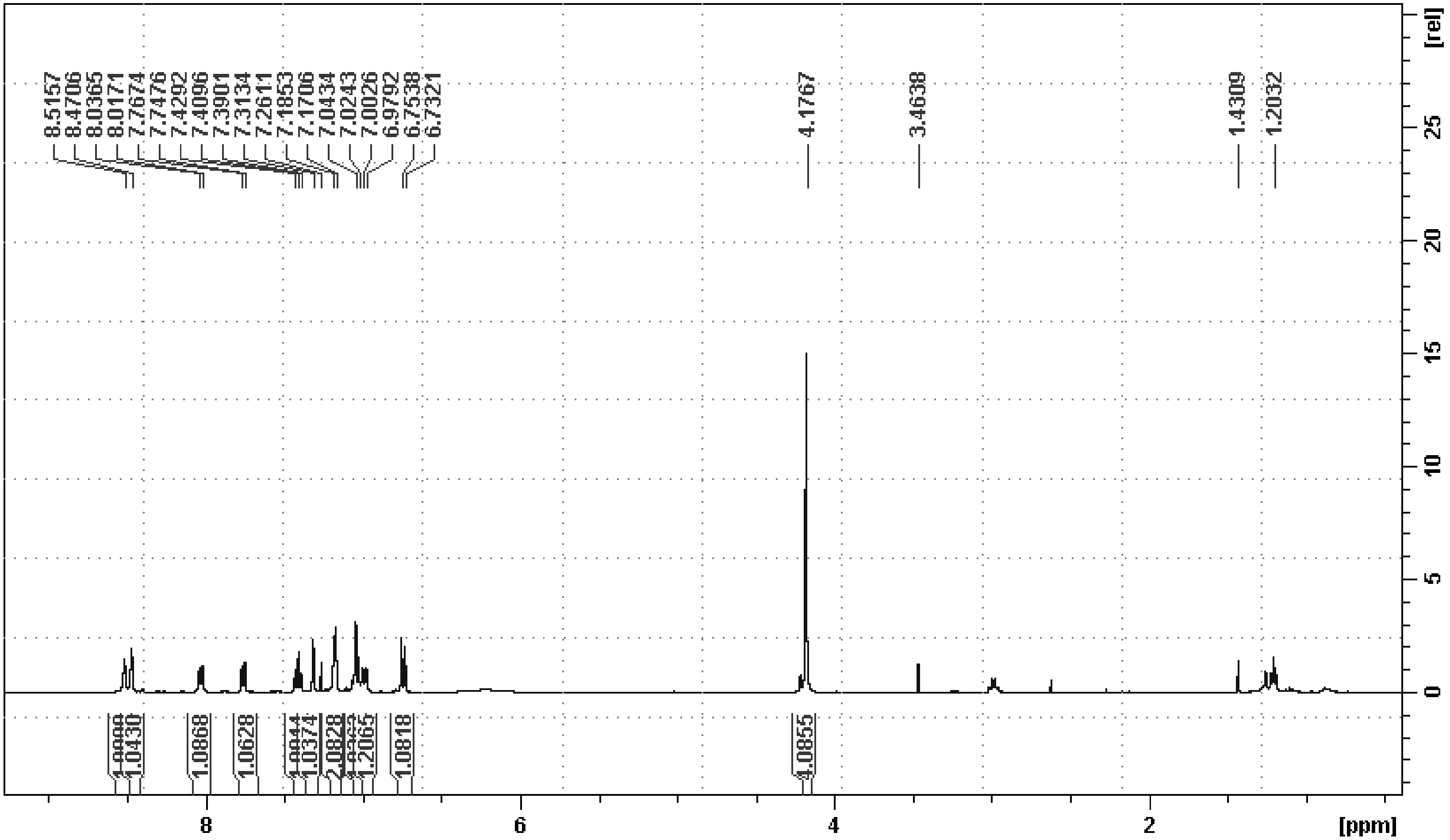

^{13}C-NMR

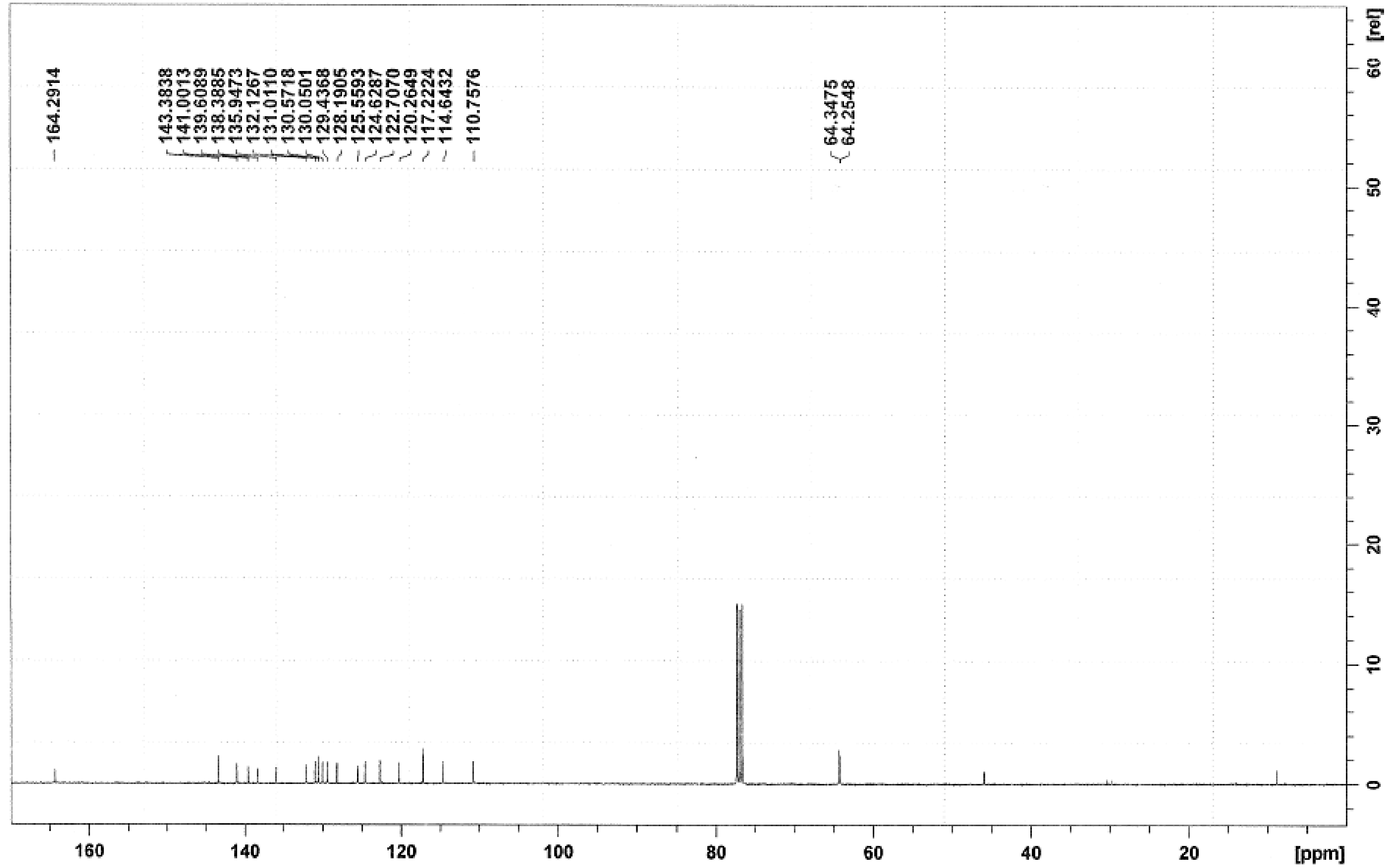

Compound **3ee**

^{1}H-NMR

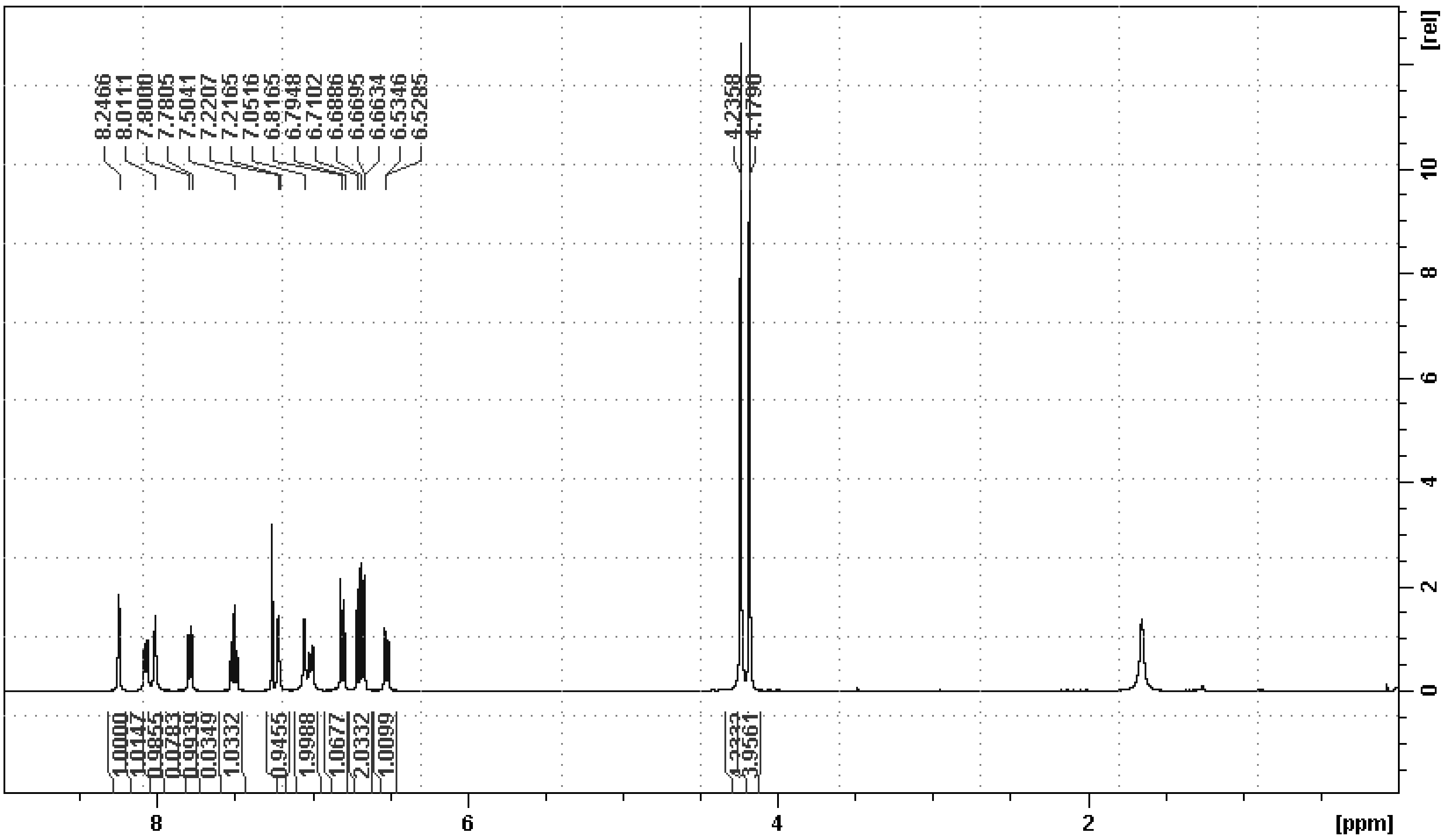

^{13}C-NMR

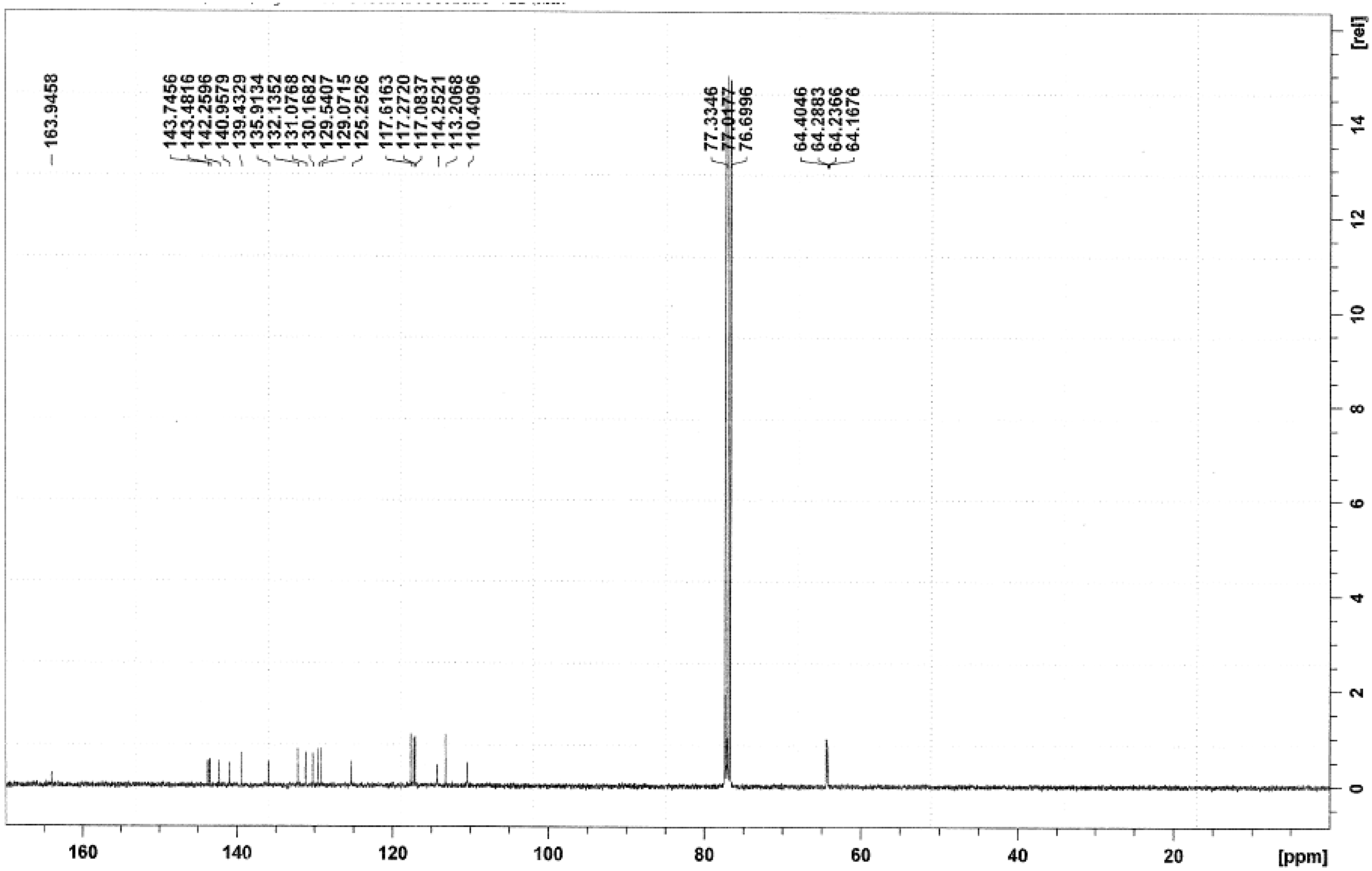

163.9458
143.7456
143.4816
142.2596
140.9579
139.4329
135.9134
132.1352
131.0768
130.1682
129.5407
129.0715
125.2526
117.6163
117.2720
117.0837
114.2521
113.2068
110.4096
77.3346
77.0177
76.6996
64.4046
64.2883
64.2366
64.1676
[rel]
[ppm]

Compound **3eg**

^{1}H-NMR

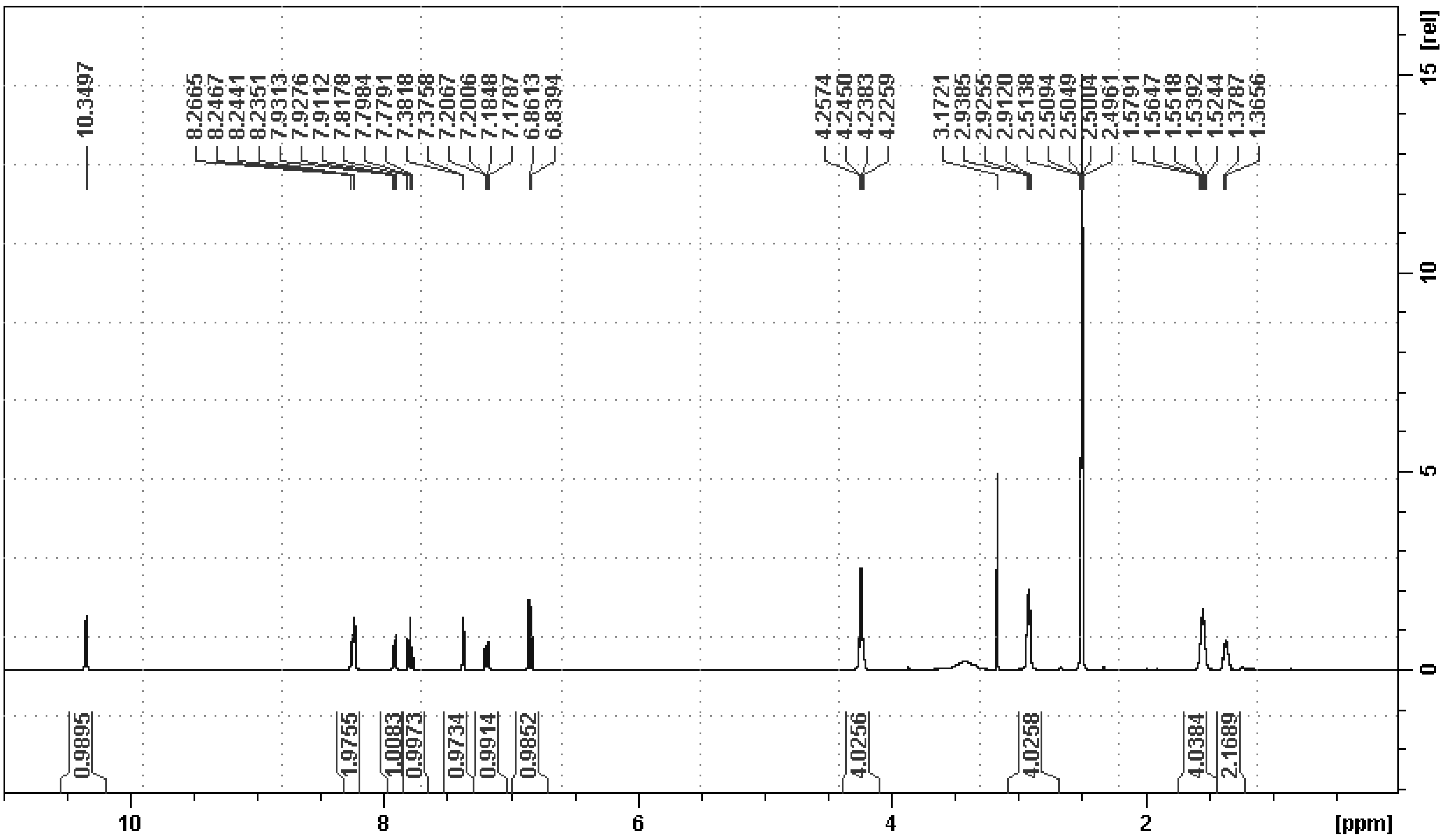

^{13}C-NMR

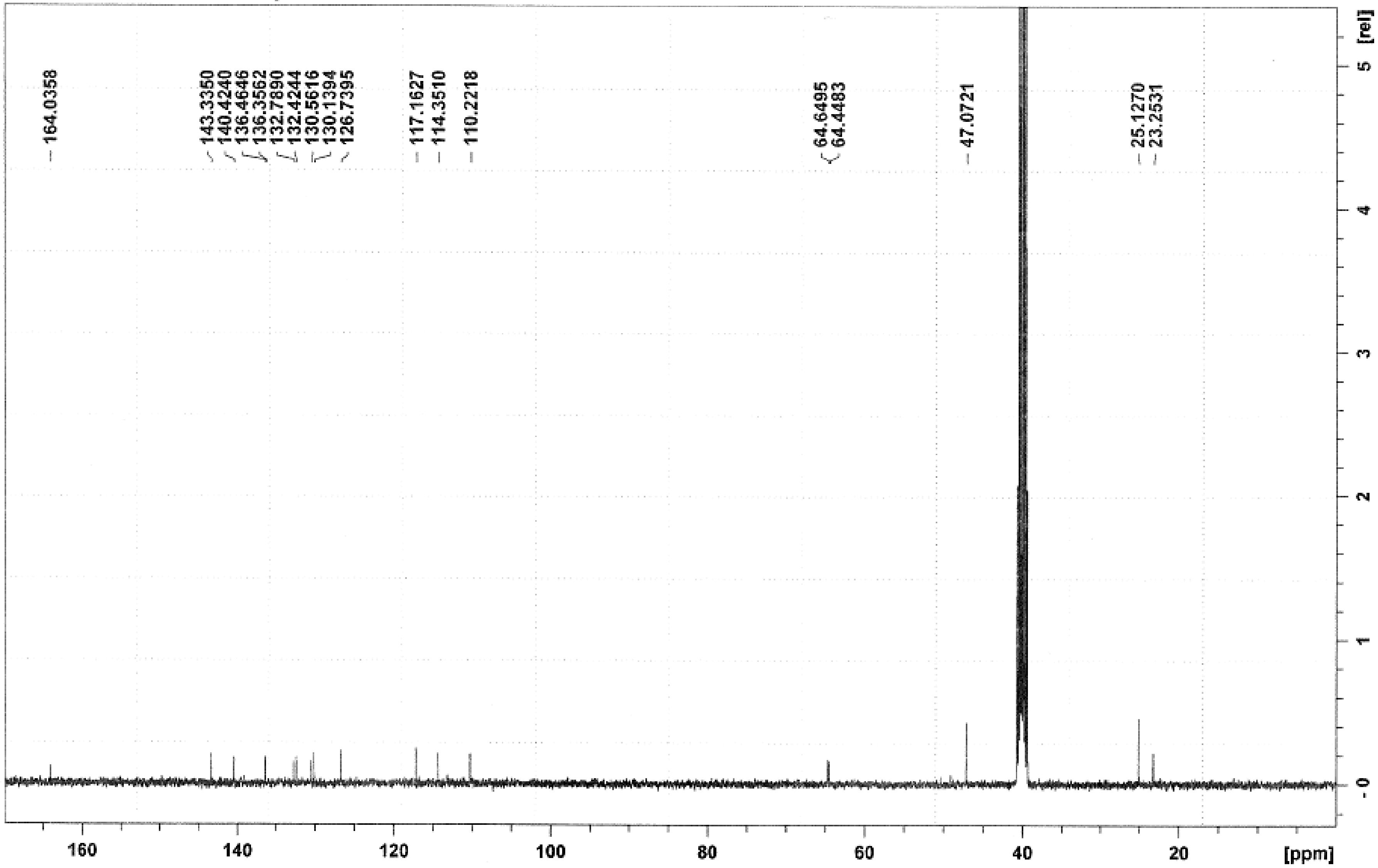

164.0358
143.3350
140.4240
136.4646
136.3562
132.7890
132.4244
130.5616
130.1394
126.7395
117.1627
114.3510
110.2218
64.6495
64.4483
47.0721
25.1270
23.2531
[rel]
[ppm]
